# Prospective association between a Mediterranean-style dietary score in childhood and cardiometabolic risk in young adults from the ALSPAC birth cohort

**DOI:** 10.1007/s00394-021-02652-7

**Published:** 2021-09-17

**Authors:** Genevieve Buckland, Caroline M. Taylor, Pauline M. Emmett, Laura Johnson, Kate Northstone

**Affiliations:** 1 Centre for Academic Child Health, Bristol Medical School, University of Bristol, Bristol, UK; 2 Centre for Exercise, Nutrition and Health Sciences, School of Policy Studies, University of Bristol, Bristol, UK; 3 Department of Population Health Sciences, Bristol Medical School, University of Bristol, Bristol, UK

**Keywords:** Mediterranean dietary pattern, Cardiometabolic risk score, Children and adolescents, ALSPAC, Prospective cohort study

## Abstract

**Purpose:**

To investigate the prospective association between a children’s relative Mediterranean-style diet score (C-rMED) in childhood and a Cardiometabolic Risk (CMR) score in adolescence/young adulthood in the Avon Longitudinal Study of Parents and Children (ALSPAC).

**Methods:**

A C-rMED was calculated at 7, 10 and 13 years from diet diary data. Anthropometric and biochemical data at 17 (*N* = 1940) and 24 years (*N* = 1961) were used to calculate CMR scores (sum of sex-specific log-transformed z-scores from triacylglycerol, HDL cholesterol, LDL cholesterol, mean arterial blood pressure, homeostatic model assessment of insulin resistance (HOMA-IR) and fat mass index (FMI)). Adjusted logistic regression models examined associations between C-rMED (categorical and 2-unit increments) and a high CMR score (≥ 80th percentile) and individual CMR components (≥ 80th percentile).

**Results:**

A high C-rMED at 13 was associated with a 32% (OR 0.68 (95% CI: 0.49, 0.94)) decreased adjusted odds of having a high CMR score at 24 years, compared to participants with a low C-rMED. No associations were evident at other ages. Tracking of the C-rMED across the three ages showed a stronger negative association between C-rMED and CMR at 24 years when children had at least two high C-rMED scores from 7 to 13 years (adjusted OR: 0.49, 95% CI: 0.29, 0.85), compared to all low scores. FMI and HOMA-IR were the main CMR components contributing to this association.

**Conclusion:**

Higher Mediterranean-style diet scores in early adolescence were associated with a better CMR profile in young adults (24 year olds). This underscores the importance of establishing healthy eating habits early in life for future cardiometabolic health.

**Supplementary Information:**

The online version contains supplementary material available at 10.1007/s00394-021-02652-7.

## Introduction

The pathogenesis of cardiometabolic diseases can begin early in life, with evidence of arterial narrowing and stiffness in childhood and fatty streaks in the coronary vessels of young adults [1]. This is linked to early onset of cardiometabolic risk (CMR) factors, such as childhood obesity, elevated blood pressure, hyperinsulinemia, dyslipidaemia and endothelial alterations [2]. CMR factors present in childhood are likely to persist into adulthood [2–4] and cardiometabolic health is associated with lifestyle habits throughout the lifespan [2, 5, 6]. Extensive research in adults has shown that diet is one of the key modifiable factors that can impact cardiometabolic diseases and their risk factors [7, 8]. However, much less is known about the influence of diet earlier in life, specifically dietary patterns, on later cardiometabolic health.

Dietary pattern analyses allow research into the combined effect of foods consumed on CMR markers and may be more easily translated into public health recommendations [9]. In recent reviews of mainly cross-sectional studies using data-driven a posteriori extracted dietary patterns and CMR factors in children, ‘unhealthy’ or ‘Western’ dietary patterns were generally associated with adverse cardiometabolic alterations in children and adolescents. Conversely ‘traditional’, ‘prudent’ and ‘health-conscious’ patterns, usually characterised by plant-based foods and fish, were related to healthier CMR profiles [9–11]. In several longitudinal studies in children, data-driven dietary patterns have been shown to be associated with CMR factors (beyond obesity) and/or markers of endothelial function and subclinical atherosclerosis [12–17].

In terms of a priori dietary patterns, the Mediterranean dietary pattern (MDP) is internationally recognised for its health benefits, above all cardiometabolic health [18, 19]. Prospective studies and randomised clinical trials in adult populations have shown strong evidence that the MDP reduces mortality and morbidity from major chronic diseases, particularly cardiovascular disease (CVD) [18–20]. This is likely due to its protective effects on developing CVD risk factors such as hypertension, insulin resistance, dyslipidaemia and overweight/obesity [19, 21, 22], as well as early markers of atherosclerosis [23]. However, few studies have researched the impact of a MDP on CMR factors in paediatric populations and, moreover, they have produced mixed findings [11]. A dietary intervention and two observational studies in children found a protective effect of the MDP against several CMR factors [24–26]. However, the MDP was not associated with a composite CMR score in a cross-sectional analysis of Finnish children in the PANIC study [27], nor was it prospectively related to individual CMR factors in the Northern Ireland Young Hearts study [17].

Composite CMR scores incorporate cardiometabolic markers such as adiposity, plasma lipids, indicators of glucose metabolism and blood pressure, and give a useful summary of overall cardiometabolic health [28]. A recent review of CMR scores in children up to 10 years identified only eight studies focusing on dietary factors [28]. In addition, findings from prospective studies using a priori indices are scarce and to our knowledge no study has examined the influence of a MDP in childhood on overall CMR in early adulthood. Understanding if the health effects of childhood dietary habits persist into adulthood is particularly important for preventative measures. The MDP, although not inherent to typical UK dietary habits, is relevant to study in relation to CMR in non-Mediterranean countries due to its renowned cardioprotective properties. Therefore, we prospectively assessed whether a dietary pattern closer to the MDP in children aged 7, 10 and 13 years was associated with a CMR score in adolescence and young adulthood (17 and 24 years) in the Avon Longitudinal Study of Parents and Children (ALSPAC).

## Methods

### Cohort description

**Fig. 1:**
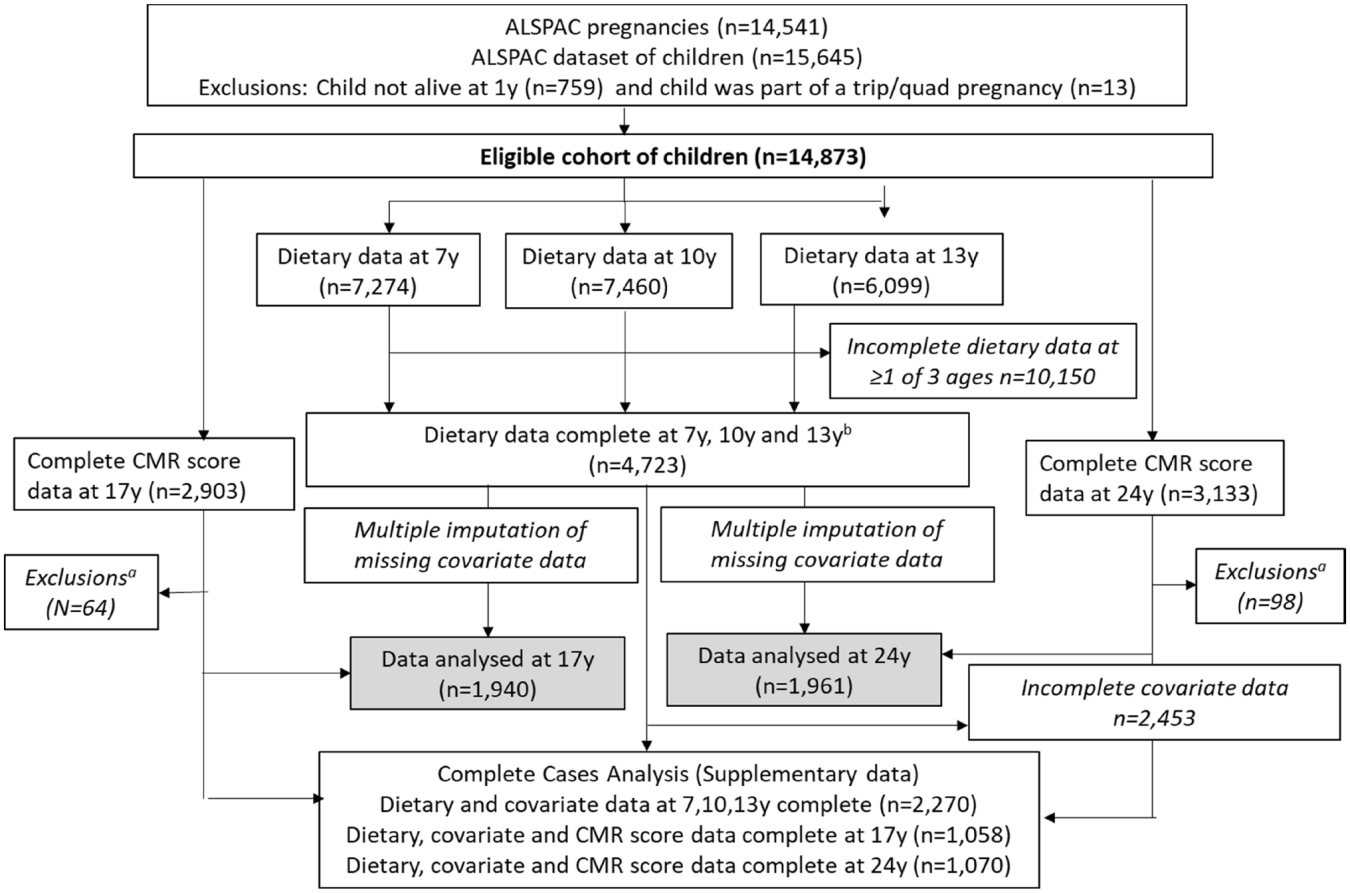
Study flow diagram for participant data from the Avon Longitudinal Study of Parents and Children (ALSPAC). The present study analyses data from participants with complete dietary data at 7, 10 and 13 years and complete data on the cardiometabolic parameters to derive the CMR score at 17 years and 24 years and uses multiple imputation for missing data in covariates. ^a^Exclusions include participants with diagnosed diabetes, on insulin treatment or fasting glucose level ≥ 7 mmol/L and subjects with extreme outliers, defined as more than 4 sd from the mean, on any of the six CMR score components. ^b^Complete dietary data refers to at least one diet diary recorded for a child at all three ages (7, 10 and 13 years). Three complete days of diet diary data were available for 88.4, 86.4 and 82.4% of children at 7, 10 and 13 years, respectively

The study participants were the index children of ALSPAC. ALSPAC is an ongoing British birth cohort established in the 1990s to investigate the determinants of health and disease across the life course [29]. Full details of the study have been reported previously [30–32] and are also available on the ALSPAC website (www.alspac.bris.ac.uk). In summary, 14,541 eligible pregnant women from the South West of England were initially enrolled into the study in 1991–1992, resulting in 13,988 children alive at 1 year. Two subsequent recruitment phases [32] in 1999 (child mean age: 7.5 years) and in 1999–2012 (child mean age: 17.8 years) resulted in a final sample of 14,873 eligible children alive at 1 year (including siblings but excluding triplet and quadruplet pregnancies for reasons of confidentiality), Fig. 1. During periodic follow-ups, extensive data have been collected from the parents and their children, primarily using questionnaires, medical records and face to face visits. Study data were collected and managed using Research Electronic Data Capture (REDCap) tools hosted at the University of Bristol [33]. REDCap is a secure, web-based software platform designed to support data capture for research studies. The study website contains details of all the data that are available through a fully searchable data dictionary and variable search tool (http://www.bristol.ac.uk/alspac/researchers/access). Ethical approval for the study was obtained from the ALSPAC Ethics and Law Committee and the Local Research Ethics Committee (http://www.bristol.ac.uk/alspac/researchers/research-ethics/) and conformed to the Declaration of Helsinki. Informed consent for the use of data collected via questionnaires and clinics was obtained for all participants, including from children when appropriate or caregivers giving consent on their behalf. Consent for biological samples was collected in accordance with the Human Tissue Act (2004).

### Dietary assessment

Children were invited to attend research clinics at the age of 7, 10 and 13 (mean age at attendance: 7.5 (sd = 0.31), 10.6 (sd = 0.22) and 13.9 (sd = 0.19) years). Prior to these clinics a 3-day diet diary was sent to them for completion, recording all food and drink consumed over two weekdays and one weekend day. This was done by the caregiver when the child was 7 years and by the children with assistance from an adult when the children were 10 and 13 years. Foods and drinks consumed were recorded using standard household measures (bowls, cups, teaspoons, dessert spoons, packet size, etc.), and included a full description of the food and the amount offered, with a separate section for description of leftovers. A short questionnaire was sent with the diaries at each age. It asked for extra details on daily foods such as types and thickness of slices of bread and fat spread used, as well as details of drinks, tea, coffee and soft drinks and included recording the volumes of cups/mugs/flasks usually used, measured at home. During the clinic visits at 10 and 13 years a nutritionist checked the diaries and questionnaires for completeness or discrepancies and clarified portion sizes.

Food and drinks described were converted into weights using the household measures or standard portion sizes relative to the age of the child based on data from the British National Diet and Nutrition Survey (NDNS) of children and young people [34] and linked to food composition tables using DIDO (Diet In Data Out). McCance and Widdowson’s British food composition data were used to calculate nutrient intakes [35]. Dietary data were then grouped according to NDNS food groupings. The average weight (g) of each food group consumed over the 3 days was used in this study. Validity of dietary reporting was calculated using an individualised method based on the ratio of energy intake to estimated energy requirement and its 95% confidence interval [36]. Further details on the ALSPAC dietary assessment methods have been published previously [37]. Data from dietary diaries were available for 7274 children at 7 years, for 7460 at 10 years and for 6099 at 13 years. 4723 had complete dietary data all three ages (Fig. 1).

### Mediterranean-style diet score

Alignment with a Mediterranean-style dietary pattern was assessed using a Children’s relative Mediterranean-style diet (C-rMED) score, which is based on the relative Mediterranean diet (rMED) score previously utilized in adults in Mediterranean and non-Mediterranean countries [38] and adapted for use in this cohort of children, as described below and detailed in Table 1. It takes into account eight dietary components: fruit (including nuts and seeds), vegetables (excluding potatoes), pulses, cereals and cereal products, fish and seafood, dairy products, meat and meat products and a lipid ratio (sum of monounsaturated fatty acids (MUFA) and polyunsaturated fatty acids (PUFA) divided by saturated fatty acids (SFA) g/day). Each component was calculated as a function of energy density (g/4184 kJ/d (g/1000 kcal/day)), using the energy density method [39]. For fruit, vegetables, cereals and dairy products, a value of 0, 1, or 2 was assigned if the children’s consumption was in the first, second, or third tertile of intake, respectively (positively scoring higher intakes). In contrast to the adult version of the rMED score, a higher consumption of dairy products was scored positively here, in accordance with the direction of scoring of dairy in the KIDMED index [40], a Mediterranean dietary score developed for use in children. For fish and seafood and legumes there were large proportions of non-consumers, therefore, a value of 0 was assigned to non-consumers and 1 and 2 points were assigned to intakes below and above the median of consumers, respectively. Olive oil was a component in the prior rMED score [41], but since olive oil was rarely consumed by the ALSPAC index children at the time of data collection we used a lipid ratio instead, which forms part of the modified Mediterranean diet score developed by Trichopoulou et al. [42]. Zero and 1 point were assigned for intakes below and above the median lipid ratio, respectively. The scoring was reversed for meat intake (positively scoring lower intakes), whereby a value of 0, 1 and 2 was assigned to the third, second or first tertile of intake. We did not include alcohol in the C-rMED since it was constructed in children (and consumption was negligible). The points received from each of the eight components were summed to give an overall C-rMED for each child at each age (7, 10 and 13 years). The possible scores ranged from 0 to 15 units, representing minimum to maximum Mediterranean-style diet. The continuous C-rMED was categorised into low (0–5), medium (6–8) and high (9–15) scores. It was also analysed per 2-unit increment to assess if more modest differences in this dietary pattern (that could relate to more easily attainable changes in dietary habits) were associated with CMR, and to evaluate the shape of the association. A combined C-rMED variable to represent tracking of the DP over time was constructed by taking into account each participants’ C-rMED category at 7, 10 and 13 years, resulting in four mutually exclusive groups: low at all ages, different category at all ages, medium category at least twice, high category at least twice.

**Table 1:** Construction of the C-rMED

Dietary components g/1000 kcal/day	Points assigned		
	0	1	2
1. Vegetables (excluding potatoes)	Tertile 1	Tertile 2	Tertile 3
2. Fruits (including nuts and seeds)			
3. Cereals and cereal products			
4. Dairy products			
5. Pulses	Non-consumers	< Median^a^	≥ Median^a^
6. Fish & Shellfish	Non-consumers	< Median^a^	≥ Median^a^
7. Monounsaturated and polyunsaturated/saturated fatty acids (ratio)	< Median	≥ Median	
8. Meat and meat products	Tertile 3	Tertile 2	Tertile 1

^a^Median calculated among consumers of this food group

### Cardiometabolic risk (CMR) factors

Cardiometabolic risk was assessed using measurements and blood samples collected by study nurses and clinic staff using standardised procedures, when the participants attended study clinics at 17.8 years (sd 0.38) and 24.5 years (sd 0.78).

#### Blood pressure

Blood pressure was measured in a seated position and resting state, using an Omron 705 IT and Omron M6 oscillometric recorder (Omron Electronic Components Europe BV) at the 17-year and 24-year clinic, respectively. Systolic blood pressure (SBP) and diastolic blood pressure (DBP) were measured twice on the right arm, using the appropriate cuff size for the upper arm circumference, and the mean of each was recorded. Mean arterial blood pressure (MAP) was then calculated using the formula: 1/3(SBP) + 2/3(DBP) [43].

#### Metabolic markers

Blood samples were taken using standard procedures and in a fasting state; participants were asked to fast overnight or at least 6–8 h prior to the clinic visit. The samples were immediately centrifuged and frozen at − 80 °C. The samples collected were assayed 3–9 months later, with no previous freeze–thawing cycles. Plasma lipids (total cholesterol, triacylglycerol, low-density lipoprotein cholesterol (LDL-c) and high-density lipoprotein cholesterol (HDL-c)) were performed according to the standard Lipid Research Clinics Protocol using enzymatic reagents for lipid determination. Glucose and insulin were used to calculate the homeostatic model assessment of insulin resistance (HOMA-IR) using the following formula: (fasting plasma glucose (mg/dl) × fasting plasma insulin (mU/L))/405 [44].

#### Body size and composition

At each age, participant height was measured to the nearest 0.1 cm using a Harpenden stadiometer (Holtain Ltd, Crymych, Pembs, UK) and weight using the Tanita Body Fat Analyser weighing scale (Tanita, West Drayton, Middlesex, UK). Waist circumference was measured to the nearest millimetre using Seca 201 body tension tape. Height and circumference were measured to the nearest millimetre, while weight was measured to the nearest 0.1 kg. Body mass index (BMI) was calculated as body mass (kg)/height (m)^2^. Fat mass (kg) was assessed at 17 and 24 years using a Lunar Prodigy Dual Emission X-ray Absorptiometry (DXA) scanner (GE Medical Systems, Madison, Wisconsin) and fat mass index (FMI) was calculated as fat mass (kg)/height (m)^2^.

### Cardiometabolic risk (CMR) score

A composite standardized continuous cardiometabolic risk (CMR) score was calculated for each participant at 17 and 24 years, in line with previous methods [28]. The CMR score included six cardiometabolic markers: FMI, HDL-c, LDL-c, triacylglycerol, MAP and HOMA-IR. Complete data on all markers were available for *n* = 2903 at 17 years and *n* = 3133 at 24 years. Participants with diagnosed diabetes (*n* = 17) or on insulin treatment (*n* = 15) and participants with fasting glucose level ≥ 7 mmol/L but who had not reported diabetes (*n* = 4 at 17 years and *n* = 14 at 24 years), were excluded from the analysis. In addition, subjects with extreme outliers (defined as more than 4 sd from the mean) on any of the six CMR score components were excluded (*n* = 42 at 17 years and *n* = 52 at 24 years).

CMR scores were calculated for participants who had complete dietary data at 7, 10 and 13 years and complete outcome data at 17 years (*n* = 1940) or 24 years (*n* = 1961). Each component measured at 17 and 24 years was log-transformed (due to right skewing of the data) and then sex-specific *z* scores were calculated to standardize the units [*z* score(component1) = (individual’s value–sex-specific sample mean)/sex-specific sample SD]. HDL-c was multiplied by − 1, to align the direction of values for increased risk with the other components. The *z* scores from the six CMR components were summed and divided by six to give the final CMR score for each participant at 17 years and at 24 years. To classify the group of participants at higher risk within this cohort, while also considering statistical power, we considered participants ≥ 80th percentile of the CMR score at increased cardiometabolic risk. The same threshold for increased risk was also used when analysing the individual CMR risk factors. In complementary analyses the CMR score was also analysed as a continuous variable.

### Covariates

Data on sex, birth weight and gestational age at birth were collected by ALSPAC staff at delivery, from medical records or from birth notifications. Maternal age at delivery was derived from maternal date of birth and child’s date of birth. Exact age (months) of study participants was recorded at all clinics. Maternal data were collected by self-completion postal questionnaires during pregnancy. Pre-pregnancy BMI of the mother was calculated from self-reported height and weight. Maternal education was reported as the highest completed out of Certificate of Secondary Education (CSE), vocational training, O-level/General Certificate of Secondary Education (qualifications obtained at 16 years of age), A-levels (qualification obtained at 18 years), University degree or higher. Maternal and paternal social class were derived using the 1991 Office of Population Censuses and Surveys (OPCS) occupation-based classification, based on the fathers/partners/mothers current or last job at 32 weeks of gestation. This resulted in standardised UK social class classifications: class I (highest), II, III–non-manual, III–manual, IV and V [45]. Maternal and paternal social class were combined to give highest family social class. Puberty timing was estimated using peak height velocity, previously calculated using a mixed effects shape-invariant growth curve model which plots a mean growth curve using data on repeated height measurements between 5 and 20 years [46]. Physical activity was assessed at 13 years using an Actigraph AM7164 2.2 accelerometer (Actigraph LLC, Fort Walton Beach, FL, USA), which children wore around their waist, at the right hip, for seven consecutive days [47]. A valid day was defined as providing data for at least 10 h per day and children were only included in the analyses if they provided at least three valid days of recording. Moderate-to-vigorous physical activity (MVPA) was calculated using the mean minutes per day in which there were > 3600 accelerometer counts per minute.

Based on previous literature on factors associated with dietary patterns and/or cardiometabolic health, the following covariates were selected a priori as potential confounders or modifiers of the association between C-rMED and CMR, and included within the multivariable logistic models as follows: sex (binary), age at corresponding dietary assessment and outcome (continuous: years), birth weight (continuous: grams), gestational age at birth (categorical: 24–36 versus ≥ 37 weeks), maternal age at delivery (categorical: < 16–24, 25–29, 30–34, ≥ 35 years), maternal pre-pregnancy BMI (continuous: kg/m^2^), mothers’ highest education level (categorical: CSE, vocational and O-level *versus* A-level or above), family highest social class (categorical: class I, II, III-non-manual, III-manual, IV and V), puberty timing (categorical: early *versus* late), MVPA (categorical: < 20 min, ≥ 20 to < 40 min, ≥ 40 to < 60 min and ≥ 60 min), validity of dietary reporting (categorical: under-reporters, plausible reporters, over-reporters), number of days dietary diary collected (categorial: 1, 2, ≥ 3 days) and total energy intake (continuous: kJ/day). The variance inflation factors for the covariates in all the different models were all ≤ 5, so the assumption of no multicollinearity was supported.

### Statistical analysis

Statistical analyses were performed using Stata version 15.1 (Stata Corporation, College Station, Texas). Of the baseline cohort of 14,873 participants alive at 1 year, 4723 had data from at least 1 diet diary at all three ages and 2270 had complete dietary and covariate data (Fig. 1). Baseline characteristics of these 2270 participants and CMR score components at 17 years (*n* = 1940) and 24 years (*n* = 1961) were compared across C-rMED categories at the three ages (7, 10 and 13 years), using proportions for categorical variables and means (sd) or medians (inter-quartile ranges (IQR)) for normal and non-parametric continuous variables, respectively. Chi-squared test were used to test differences between categorical variables and Kruskal–Wallis test for continuous variables. The same statistical tests were used to compare participants with complete dietary and covariate data at all three ages (*n* = 2270) to participants with missing dietary data at one (*n* = 2344), two (*n* = 1965) or all three (*n* = 5837) time points and incomplete covariate data (*n* = 12,603 in total; 85% of total eligible cohort). The correlation between the continuous C-rMED score between 7, 10 and 13 years was measured using partial Pearson correlation coefficient adjusted for dietary misreporting. The correlation between the continuous CMR score at 17 and 24 years was also assessed in the 1159 participants with data on both outcomes. In line with previous research on correlation of dietary patterns across time [15], a coefficient of < 0.30 was the cut-off point applied to indicate low correlations, between 0.30 and 0.59 for moderate correlations and ≥ 0.60 as high correlations.

The association between the C-rMED (categorical and per 2-unit increment at 7, 10 and 13 years and C-rMED tracking from 7 to 13 years) and a high CMR score at 17 years and 24 years was assessed using unadjusted and adjusted multivariable logistic regression models for each exposure and outcome combination. A trend test for the categorical C-rMED was carried out by analysing the C-rMED as a continuous variable with the C-rMED categories scored as 1–3. The likelihood ratio test was used to assess the shape of the association [48] by comparing the model with a categorical C-rMED variable to the respective model with a continuous C-rMED variable. The CMR score was assessed as a binary outcome (< 80th percentile: reference, and ≥ 80th percentile). Separate multivariable logistic regression models were similarly applied to examine the odds of being ≥ 80th percentile for each individual log-transformed CMR score component (FMI, HDL-c, LDL-c, triacylglycerol, MAP and HOMA-IR) and additional CMR factors (BMI, waist circumference, SPB, DBP, total cholesterol, insulin and glucose) in relation to a 2-unit increment in C-rMED at each age.

### Sensitivity analyses

In additional anayses, adjusted linear regression models were used to assess the association between the C-rMED and CMR score as a continuous variable in complete-case analyses (*n* = 1058 for outcome at 17 years and *n* = 1070 for outcome at 24 years), with ß coefficients and 95% confidence intervals (CI) presented in supplementary material. The relative importance of each C-rMED component for predicting high CMR was also assessed; each C-rMED component was alternatively subtracted from the original score (e.g. C-rMED without vegetables or without pulses). The OR and 95% CI for ≥ 80th CMR score percentile was calculated for a 2-unit increment in each C-rMED score minus one component, and the percentage change in OR compared to the complete C-rMED was calculated. Results were presented for both sexes together, as there was no evidence that the relation between the C-rMED and CMR score or individual CMR components was modified by sex in any of the models (likelihood ratio (lr) test comparing models with and without an interaction term between the C-rMED and sex variables resulted in *p* values > 0.10).

### Missing data and multiple imputation

All multivariable logistic regression models included the 13 different covariates listed previously and 45% of participants had missing data on at least one of the covariates and the largest amount of missing data was observed for PA with 21% at 17 and 24 years. In ALSPAC, participants from lower socioeconomic groups are less likely to be retained in the study. Therefore, to minimize attrition bias and increase efficiency and precision of estimates [49] we imputed missing covariate data using multiple imputation with chained equations (ICE) in Stata using the ‘ICE’ command. Twenty stacked datasets were generated and used in the final analyses. The variables included in the imputation models were all of those included in the final regression models and additional auxiliary variables (Family Adversity Index [50], parity, and child’s BMI at time of dietary data collection) which also strongly predicted missingness in the covariates. Thus, the basic assumption underlying multiple imputation that data were ‘missing at random’ [51] was supported, since missingness could be explained by the auxiliary variables and other covariates in the model. Separate imputed datasets were created for the participants with complete dietary and CMR data at 17 years (*n* = 1940) and at 24 years (*n* = 1961). The results from the regression analysis using the imputed datasets are presented in the main article, while the supplementary material outlines the results from complete-case analyses.

## Results

The study sample used in the final imputed dataset and complete-case dataset is shown in Fig. 1 and Appendix I in Supplementary material. The main analysis sample included 1940 (50% female) participants with complete C-rMED at all ages and CMR score data at 17 years, of which 388 (20%) participants were classified as being in the high CMR group. A total of 1961 (57% female) participants had complete C-rMED and CMR score data at 24 years, of which 392 (20%) participants were classified as being in the high CMR group.

The percentage of missing data on each covariate in the analyses at 17 years and 24 years ranged from 0 to 21% and is detailed in Appendix II in Supplementary material. Compared to participants with missing dietary and covariate data, those with complete dietary and covariate data at all three ages (*n* = 2270) were more likely to be female, had a lower BMI at 10 years, a higher C-rMED at all ages, a better metabolic profile at 17 years and had a higher social class (Appendix III in Supplementary material). The baseline characteristics of ALSPAC participants with imputed and observed data (Appendix IV of Supplementary material) was very similar.

The baseline characteristics and CMR factors (at 17 and 24 years) for participants with complete dietary and covariate data (*n* = 2270) according to category of C-rMED at 7, 10 and 13 years is described in Table 2. In general, participants in the high C-rMED group at all three ages had mothers who were older and with a higher level of education and family social class, compared to those in the low c-rMED group. Participants in the high C-rMED group at 10 and 13 years also had a lower total energy intake than those in the low group. Participants in the high compared to the low C-rMED group at 13 years had a lower FMI and metabolic risk measures at 24 years.

**Table 2:** Characteristics of the study sample with complete dietary and covariate data, according to the low and high categories of the children’s relative Mediterranean diet score (C-rMED) at 7, 10 and 13 years

Characteristics of the ALSPAC index children	C-rMED at 7 years^a^				C-rMED at 10 years^a^				C-rMED at 13 years^a^			
	Low	Medium	High	*P* value	Low	Medium	High	*P* value	Low	Medium	High	*P* value
Baseline Characteristics, *n* (total *n* = 2270)	690	1023	557		692	1042	536		754	1002	513	
Age^b^, y	7.5 ± 0.2^d^	7.5 ± 0.1	7.5 ± 0.1	0.787	10.6 ± 0.2	10.6 ± 0.2	10.6 ± 0.2	0.849	13.8 ± 0.2	13.8 ± 0.2	13.8 ± 0.2	0.455
Female	54.2^e^	54.5	57.5	0.454	52.6	55.2	58.2	0.212	49.7	54.6	64.0	< 0.001
BMI^c^, kg/m^2^	16.1 ± 2.0	16.1 ± 2.0	16.0 ± 1.8	0.773	18.1 ± 3.1	17.9 ± 2.9	17.9 ± 2.8	0.242	20.1 ± 3.4	20.1 ± 3.2	20.1 ± 3.2	0.916
Puberty timing, early	48.8	49.5	53.4	0.240	51.8	48.7	51.3	0.419	50.0	50.9	49.4	0.851
Maternal age at delivery, ≥ 30 years	45.4	48.1	57.6	< 0.001	42.8	50.3	57.1	< 0.001	45.9	50.9	52.5	0.037
Maternal pre-pregnancy BMI, ≥ 25 kg/m^2^	19.4	17.3	14.7	0.093	19.7	16.7	15.5	0.124	19.8	16.6	15.2	0.075
Maternal education, up to A-level / Degree	42.3	49.1	65.4	< 0.001	40.3	52.6	61.8	< 0.001	41.1	52.1	63.4	< 0.001
Highest household social class, grade I & II	26.9	32.3	42.6	< 0.001	29.1	31.4	42.0	< 0.001	27.1	34.5	39.5	< 0.001
Total energy, kJ/day	7130 ± 1275	7234 ± 1279	7150 ± 1215	0.239	7961 ± 1648	7880 ± 1472	7742 ± 1461	0.020	8502 ± 2175	8306 ± 2140	7898 ± 1925	< 0.001
Diet diaries, < 3 days	14.8	11.4	7.9	0.001	18.6	13.2	7.7	< 0.001	22.9	14.7	14.6	< 0.001
CMR factors at 17 years, *n* (total *n* = 1940)	594	855	491		566	911	463		661	851	428	
Fat mass index, kg/m^2^	5.4 (3.2–7.9)^f^	5.6 (3.1–7.7)	5.0 (3.0–7.3)	0.102	5.4 (2.9–7.8)	5.4 (3.3–7.7)	5.2 (3.1–7.6)	0.475	5.3 (2.9–7.9)	5.5 (3.2–7.8)	5.2 (3.4–7.2)	0.565
HDL cholesterol, mmol/L	1.2 (1.1–1.5)	1.2 (1.1–1.4)	1.3 (1.1–1.5)	0.134	1.2 (1.0–1.4)	1.2 (1.1–1.5)	1.3 (1.1–1.5)	0.474	1.2 (1.1–1.5)	1.2 (1.1–1.4)	1.3 (1.1–1.5)	0.060
LDL cholesterol, mmol/L	2.1 (1.7–2.5)	2.1 (1.7–2.5)	2.0 (1.6–2.4)	0.088	2.0 (1.7–2.5)	2.1 (1.7–2.5)	2.0 (1.7–2.5)	0.937	2.0 (1.6–1.4)	2.0 (1.6–2.5)	2.0 (1.7–2.4)	0.824
Triacylglycerol, mmol/L	0.7 (0.6–0.9)	0.8 (0.6–1.0)	0.8 (0.6–1.0)	0.616	0.7 (0.6–1.0)	0.7 (0.6–1.0)	0.8 (0.6–0.9)	0.906	0.7 (0.6–1.0)	0.7 (0.6–1.0)	0.8 (0.6–1.0)	0.868
Mean arterial blood pressure, mmHg^g^	81.0 (77.6–85.0)	80.8 (77.2–84.6)	80.6 (77.0–84.8)	0.511	80.7 (77.4–85.0)	81.0 (77.4–84.9)	80.6 (76.8–84.2)	0.362	81.2 (77.8–85.2)	81.2 (77.4–84.9)	79.4 (76.4–83.8)	< 0.001
HOMA-IR^h^	1.4 (1.0–2.0)	1.5 (1.1–2.1)	1.4 (1.0–1.9)	0.663	1.4 (1.1–2.0)	1.5 (1.1–2.1)	1.5 (1.0–2.0)	0.460	1.5 (1.1–2.1)	1.5 (1.1–2.1)	1.4 (1.0–1.9)	0.406
CMR factors at 24 years, *n* (total *n* = 1961)	574	890	497		575	878	508		623	882	456	
Fat mass index, kg/m^2^	7.0 (5.4–9.6)	6.8 (5.3–9.1)	6.6 (5.0–8.6)	0.043	7.1 (5.2–9.7)	6.8 (5.3–9.0)	6.6 (5.2–8.4)	0.053	7.1 (5.3–9.9)	6.7 (5.2–8.9)	6.6 (5.2–8.4)	0.026
HDL cholesterol, mmol/L	1.5 (1.2–1.8)	1.5 91.3–1.8)	1.6 (1.3–1.9)	0.012	1.5 (1.3–1.8)	1.5 (1.3–1.8)	1.6 (1.3, 1.9)	0.003	1.5 (1.2–1.8)	1.5 (1.3–1.8)	1.6 (1.3–1.8)	< 0.001
LDL cholesterol, mmol/L	2.4 (1.9–2.9)	1.6 (1.3–1.9)	2.3 (1.9–2.9)	0.133	2.4 (2.0–2.9)	2.3 (1.9–2.9)	2.3 (1.9–2.8)	0.259	2.4 (2.0–2.9)	2.3 (1.9–2.9)	2.3 (1.9–2.9)	0.165
Triacylglycerol, mmol/L	0.8 (0.6–1.1)	0.8 (0.6–1.1)	0.8 (0.7–1.1)	0.870	0.8 (0.7–1.2)	0.8 (0.7–1.1)	0.8 (0.6–1.1)	0.615	0.8 (0.7–1.1)	0.8 (0.7–1.2)	0.8 (0.6–1.1)	0.034
Mean arterial blood pressure, mmHg^g^	83.4 (78.3–88.7)	82.2 (77.6–87.8)	81.9 (77.0–87.8)	0.021	82.6 (77.9–88.3)	82.8 (77.8, 88.1)	81.8 (77.2–87.8)	0.273	83.2 (78.2–89.0)	82.5 (77.7–87.8)	81.3 (76.8–87.0)	0.010
HOMA-IR^h^	1.8 (1.2–2.5)	1.7 (1.2–2.5)	1.6 (1.2–2.4)	0.228	1.7 (1.2–2.6)	1.7 (1.2–2.4)	1.7 (1.2–2.3)	0.480	1.8 (1.2–2.6)	1.7 (1.2–2.4)	1.6 (1.1–2.4)	0.028

*P* values derived from chi-squared tests for categorical variables and Kruskal–Wallis test for continuous variables

*C-rMED* children's relative Mediterranean diet score, *CMR* Cardiometabolic risk factors

^a^C-rMED includes eight food components and scores subjects from 0 to 15 (low (0–5), medium (6–8) and high (9–15)), categories chosen to achieve relatively comparative numbers in low and high groups

^b^Age refers to age at each point of dietary data collection (7, 10, 13 years)

^c^BMI corresponds to measurements taken at each time point of dietary data collection

^d^Mean ± SD (all such values)

^e^Percentage (all such values)

^f^Median and inter-quartile range (all such values)

^g^Mean arterial blood pressure calculated as (2 × (diastolic blood pressure)) + (systolic blood pressure)/3

^h^HOMA-IR; Homeostatic Model Assessment of Insulin Resistance, calculated as (fasting plasma glucose (mgdl) × fasting plasma insulin(mU/L))/405

The correlation coefficient for continuous C-rMED between 7 and 10 years was 0.35 (95% CI: 0.32, 0.38), between 7 and 13 years it was 0.30 (95% CI: 0.28, 0.33) and between 10 and 13 years it was 0.34 (95% CI 0.31, 0.37), indicating moderate correlation of this dietary pattern throughout this period. The correlation coefficient for the continuous CMR score at 17 and 24 years was 0.57 (95% CI 0.52, 0.61), indicating a moderate-to-high correlation of CMR score between these ages.

Multivariable logistic regression models showed that the C-rMED, categorical and continuous, at 7, 10 and 13 years was not associated with a high CMR score at 17 years (Table 3). There was also no evidence of an association between the C-rMED, categorical or continuous, at 7 and 10 years and CMR score at 24 years. However, compared to being in the low C-rMED group at 13 years, being in the medium C-rMED group reduced the odds of having a high CMR score (≥ 80th percentile) by 27% (OR 95% CI 5–44%), and being in the high C-rMED group reduced the odds of having a high CMR score by 32% (OR 95% CI 4–51%) *p* trend 0.013; each 2-unit increment in C-rMED corresponded to a OR 0.88 (95% CI 0.80, 0.98) *p* value = 0.016 for a high CMR score. In sensitivity analyses, additional adjustment by baseline BMI did not alter this association (OR 0.69 (95% CI 0.49, 0.98) for high versus low C-rMED at 13 years, *p* trend 0.029).

**Table 3:** Unadjusted and adjusted OR and 95% CI for the association between the children’s relative Mediterranean diet score (C-rMED) at 7, 10 and 13 years and scoring ≥ 80th percentile on cardiometabolic risk score at 17 and 24 years, using imputed datasets

Children's relative Mediterranean diet score (C-rMED)^a^	CMR score (≥ 80th percentile) at 17 years (*n* = 1940)					CMR Score (≥ 80th percentile) at 24 years (1961)				
		Unadjusted		Adjusted^b^			Unadjusted		Adjusted^b^	
	*N*	OR (95%CI)	*P* trend	OR (95% CI)	*P* trend	*N*	OR (95% )	*P* trend	OR (95% CI)	*P* trend
C-rMED at 7 years										
Low	594	Reference		Reference		574	Reference		Reference	
Medium	855	0.99 (0.77, 1.29)		1.07 (0.82, 1.41)		890	0.79 (0.61, 1.02)		0.83 (0.63, 1.09)	
High	491	0.74 (0.54, 1.01)	0.079	0.85 (0.61, 1.18)	0.392	497	0.78 (0.57, 1.05)	0.086	0.89 (0.64, 1.10)	0.414
Per 2-unit increment	1940	0.89 (0.81, 0.98)	0.020	0.93 (0.84, 1.03)	0.154	1961	0.92 (0.84, 1.01)	0.097	0.97 (0.87, 1.07)	0.490
C-rMED at 10 years										
Low	566	Reference		Reference		575	Reference		Reference	
Medium	911	1.06 (0.81, 1.38)		1.14 (0.87, 1.50)		878	0.86 (0.66, 1.11)		0.91 (0.69, 1.19)	
High	463	0.87 (0.63, 1.20)	0.445	1.00 (0.71, 1.40)	0.926	508	0.70 (0.51, 0.96)	0.025	0.79 (0.57, 1.10)	0.169
Per 2-unit increment	1940	0.96 (0.87, 1.06)	0.428	1.01 (0.91, 1.11)	0.890	1961	0.89 (0.91, 0.98)	0.015	0.92 (0.84, 1.02)	0.127
C-rMED at 13 years										
Low	661	Reference		Reference		623	Reference		Reference	
Medium	851	0.81 (0.63, 1.05)		0.86 (0.66, 1.12)		882	0.67 (0.52, 0.86)		0.73 (0.56, 0.95)	
High	428	0.68 (0.49, 0.93)	0.014	0.80 (0.57, 1.11)	0.157	456	0.59 (0.43, 0.81)	0.002	0.68 (0.49, 0.94)	0.013
Per 2-unit increment	1940	0.90 (0.81, 0.99)	0.035	0.95 (0.86, 1.05)	0.306	1961	0.84 (0.77, 0.93)	0.001	0.88 (0.80, 0.98)	0.016
C-rMED tracking 7–10-13 years^c^										
C-rMED low all ages	153	Reference		Reference		134	Reference		Reference	
C-rMED mixed across ages	639	0.89 (0.57, 1.39)		0.97 (0.61, 1.53)		645	0.85 (0.55, 1.03)		0.85 (0.54, 1.36)	
C-rMED medium at least twice	833	0.89 (0.57, 1.38)		0.99 (0.63, 1.55)		843	0.58 (0.38, 0.90)		0.60 (0.38, 0.95)	
C-rMED high at least twice	315	0.69 (0.42, 1.14)	0.165	0.85 (0.50, 1.45)	0.619	339	0.42 (0.25, 0.70)	< 0.001	0.49 (0.29, 0.85)	< 0.001

*C-rMED* Children's relative Mediterranean diet score, *CMR score* Cardiometabolic risk score

^a^C-rMED includes eight food components and scores subjects from 0 to 15 (low (0–5), medium (6–8) and high (9–15))

^b^Adjusted: Multivariable regression model adjusted for sex, age at dietary data collection, number of days diet diary collected, dietary misreporting, birthweight, gestational age, puberty stage, physical activity at 13 years, pre-pregnancy BMI of mother, age of mother at delivery, mother's highest education level, highest family social class

^c^C-rMED medium at least twice includes the following combination of C-rMED scores across any of the three age groups (medium + medium + low or medium + medium + medium or medium + medium + high). C-rMED high at least twice includes the following combination of C-rMED scores across any of the three age groups (high + high + low or high + high + medium or high + high + high)

The likelihood ratio test comparing the categorical C-rMED with the continuous C-rMED supported the assumption that the association was linear (*p* > 0.05). Sex-specific results for the association between the C-rMED and CMR score are presented in Appendix V in Supplementary material, however, there was no indication of that the association was modified by sex in any of the models (lrtest *p* values > 0.10). The combined C-rMED tracking variable (taking into account C-rMED categories at 7, 10 and 13 years) was also associated with the CMR score at 24 years (*p* trend < 0.001); participants categorised as being in the medium C-rMED group at least twice throughout this period were 40% (OR 95% CI 5–62%) less likely to have a high CMR score at 24 years, while being in the high C-rMED group at least twice decreased the odds by 51% (OR 95% CI 15–71%), in both cases compared to being in the low C-rMED group at all ages (Table 3). The results from a complete-case analysis (Appendix VI in Supplementary material) were very similar, although an association between the C-rMED at 10 years and CMR at 24 years was also evident. When the complete-case analysis was re-analysed with the CMR score as a continuous variable (per unit increment), a similar negative association was observed between the C-rMED at 13 years (per 2-unit increment) and CMR score at 24 years (Appendix VII in Supplementary material).

The adjusted associations for being ≥ 80th percentile for individual CMR factors (anthropometrics, blood lipids, blood pressure and glucose metabolism) at 17 year and 24 years in relation to a 2-unit increment in C-rMED at 7, 10 and 13 years are detailed in Table 4. A 2-unit increase in C-rMED at 13 years was associated with lower odds of being ≥ 80th percentile for FMI, insulin and HOMA-IR at 17 (*p* < 0.01). There was decreased odds of high DBP at 17 years (OR 0.89 (95% CI 0.80, 0.98) for each 2-unit increment in C-rMED at 7 years. A 2-unit increase in C-rMED at 13 years was negatively associated with being ≥ 80th percentile of all anthropometric measures (BMI, FMI and waist circumference) at 24 years (*p* < 0.01), with the greatest reduction in odds for FMI (OR 0.81 (95% CI 0.73, 0.90) per 2-unit increment). The C-rMED at 13 years was also associated with better glucose metabolism at 24 years; a 2-unit increment in C-rMED resulted in 12% decreased odds (*p* value = 0.010) of being ≥ 80th percentile for HOMA-IR and 16% decreased odds (*p* value = 0.001) of being ≥ 80th percentile for insulin. There was no evidence of associations between the C-rMED and remaining CMR items at 17 and 24 years. The complete-case analysis produced similar results (Appendix VIII in Supplementary material).

**Table 4:** Adjusted OR and 95% CI for the association between the children’s relative Mediterranean diet score (C-rMED) at 7, 10 and 13 years and individual cardiometabolic risk factors at 17 and 24 years, using imputed datasets

CMR factors: Odds of being ≥ 80th percentile^a^	CMR Factors at 17y (*n* = 1940)			CMR Factors at 24y (*n* = 1961)		
	+ 2-unit C-rMED 7 y	+ 2-unit C-rMED 10 y	+ 2-unit C-rMED 13 y	+ 2-unit C-rMED 7 y	+ 2-unit C-rMED 10 y	+ 2-unit C-rMED 13 y
	OR (95% CI)^b^	OR (95% CI)^b^	OR (95% CI)^b^	OR (95% CI)^b^	OR (95% CI)^b^	OR (95% CI)^b^
Anthropometric						
Body mass index	0.95 (0.85,1.05)	1.00 (0.90, 1.16)	0.95 (0.85, 1.05)	0.92 (0.83, 1.02)	0.94 (0.84, 1.04)	0.83 (0.75, 0.92)
Fat mass index^c^	0.92 (0.82, 1.02)	0.95 (0.85, 1.07)	0.88 (0.79, 0.98)	0.95 (0.85, 1.05)	0.91 (0.81, 1.01)	0.81 (0.73, 0.90)
Waist circumference	N/A	N/A	N/A	0.90 (0.81, 1.00)	0.89 (0.81, 0.99)	0.87 (0.79, 0.97)
Blood lipids						
Total cholesterol	0.92 (0.83, 1.01)	1.04 (0.94, 1.14)	0.95 (0.86, 1.05)	1.07 (0.97, 1.18)	1.04 (0.94, 1.14)	1.02 (0.92, 1.12)
HDL cholesterol^c^	0.98 (0.88, 1.08)	0.96 (0.86, 1.06)	0.97 (0.87, 1.07)	0.93 (0.84, 1.03)	0.98 (0.90, 1.08)	0.94 (0.85, 1.03)
LDL cholesterol^c^	0.98 (0.89, 1.08)	1.01 (0.92, 1.12)	1.00 (0.91, 1.11)	1.06 (0.96, 1.17)	0.99 (0.90, 1.09)	0.97 (0.88, 1.06)
Triacylglycerol^c^	1.05 (0.95, 1.15)	1.05 (0.95,1.15)	0.99 (0.90, 1.09)	0.99 (0.90, 1.09)	0.98 (0.89, 1.08)	0.99 (0.90, 1.09)
Blood pressure						
Systolic BP	1.03 (0.92, 1.14)	1.07 (0.96, 1.19)	1.02 (0.91, 1.13)	0.93 (0.84, 1.03)	0.98 (0.88, 1.09)	0.95 (0.86, 1.04)
Diastolic BP	0.89 (0.80, 0.98)	0.99 (0.90, 1.10)	0.99 (0.89, 1.09)	0.97 (0.88, 1.07)	1.03 (0.93,1.13)	0.94 (0.85, 1.05)
Mean arterial BP^c^	0.93 (0.84, 1.03)	0.95 (0.86, 1.05)	0.98 (0.88, 1.08)	0.98 (0.88, 1.08)	1.02 (0.92, 1.12)	0.94 (0.85, 1.04)
Glucose metabolism						
Insulin	0.99 (0.90, 1.09)	1.01 (0.92, 1.12)	0.87 (0.79, 0.97)	1.00 (0.90, 1.10)	0.95 (0.86, 1.05)	0.84 (0.76, 0.93)
Glucose	1.04 (0.94, 1.15)	1.11 (1.01, 1.23)	1.02 (0.92, 1.13)	1.06 (0.96, 1.17)	1.04 (0.94, 1.15)	0.94 (0.85, 1.04)
HOMA-IR^c^	1.00 (0.91, 1.11)	1.00 (0.90, 1.10)	0.88 (0.79, 0.97)	0.99 (0.89, 1.09)	0.94 (0.85, 1.04)	0.88 (0.80, 0.97)

*C-rMED* children's relative Mediterranean diet score (+ 2-unit increments relating to modest changes in this dietary pattern), *CMR score* Cardiometabolic Risk Score, *HOMA-IR* Homeostatic Model Assessment of Insulin Resistance, *BP* Blood Pressure, *HDL cholesterol* High-density lipoprotein cholesterol, *LDL cholesterol* low-density lipoprotein cholesterol

^a^Odds of being above the 80th percentile except for HDL cholesterol, which is odds of being below the 20th percentile

^b^Odds Ratios (OR) derived from multivariable logistic regression models adjusted for sex, age at dietary data collection, number of days diet diary collected, dietary misreporting, birthweight, gestational age, puberty stage, physical activity at 13 years, pre-pregnancy BMI of mother, age of mother at delivery, mother's highest education level, highest family social class

^c^Cardiometabolic parameters included in the Cardiometabolic Risk Score

**Fig. 2:**
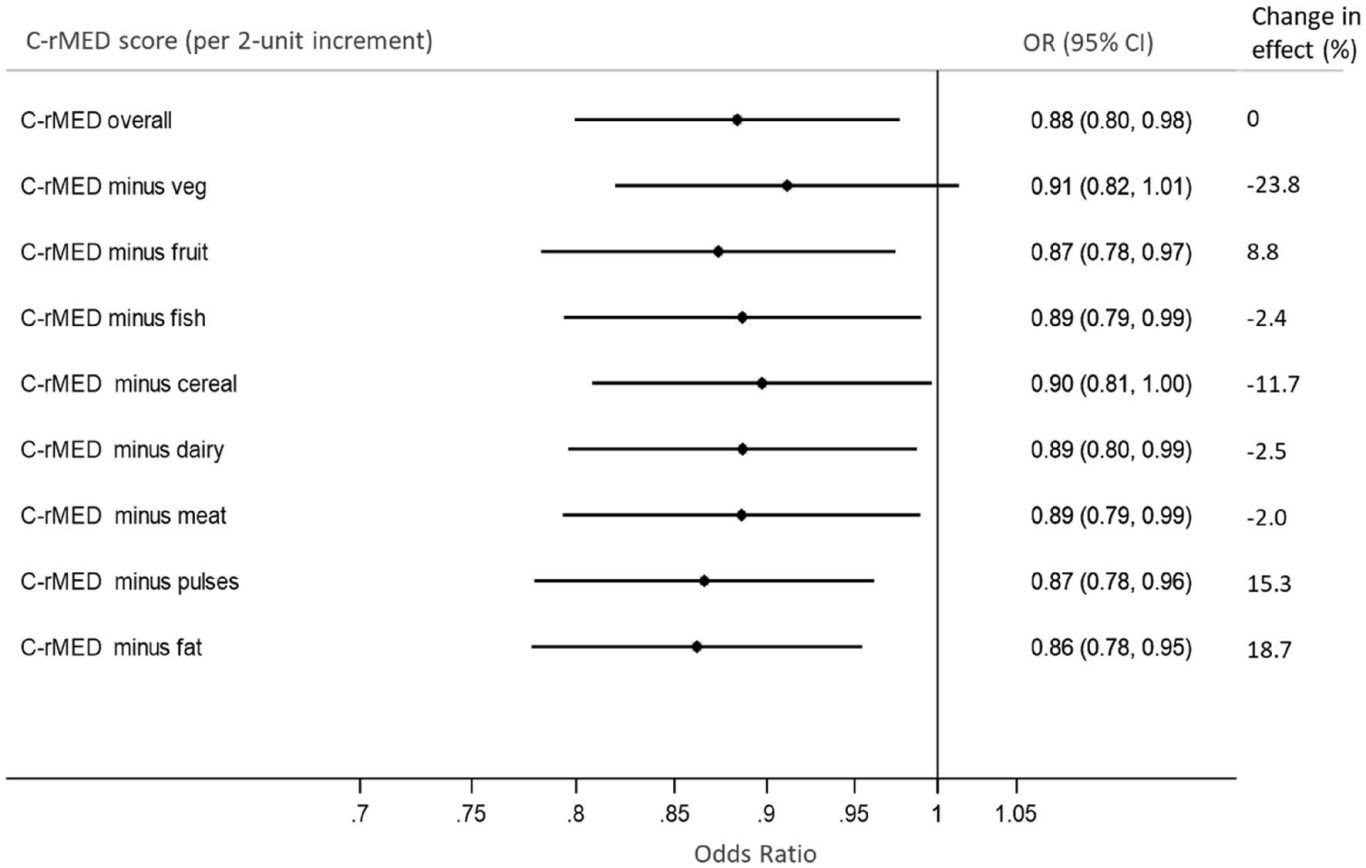
Adjusted association between a high CMR score at 24 years and a 2-unit increase in the children’s relative Mediterranean Diet Score (C-rMED) measured at 13 years, while alternatively subtracting each component of the C-rMED, using imputed datasets

Alternatively subtracting each of the eight components from the rMDS in turn showed that vegetables and cereals attenuated the association between C-rMED at 13 years and CMR score at 24 years most (Fig. 2): the percentage decrease in the association between a 2-unit increase in C-rMED and high CMR score after excluding vegetables was − 23.8% and after excluding cereals was − 11.7%. Point estimates ranged from 0.86 to 0.91 and 95% confidence intervals were overlapping suggesting that no single food entirely explains the association observed for the whole dietary pattern.

## Discussion

Having a higher Mediterranean-style diet score at 13 years was associated with a 32% reduction in the likelihood of having a high CMR score at 24 years in this cohort. Each 2-unit increase in the C-rMED resulted in 12% reduced odds of a high CMR score. Maintaining a Mediterranean-style diet throughout childhood was associated with an even greater protective effect: participants who mostly had a high C-rMED compared to always having a low C-rMED from 7 to 13 years were 51% less likely to have a high CMR score at 24 years. Of public health importance is that a 2-unit increment in the C-rMED, which would translate as a small shift in dietary habits towards a more Mediterranean-style diet, was related to better overall cardiometabolic health. The association was largely driven by the impact of the C-rMED on reducing adiposity and improving glucose metabolism. Our findings are particularly salient given that several large prospective studies have found that these conventional childhood CMR factors are predictive of early markers of atherosclerosis in young adults [4, 14]. In addition, observational studies have reported an inverse association between MDP scores in childhood and indicators of arterial stiffness in children [52] and young adults [17, 53].

To our knowledge, this is the first prospective study to show that a MDP in adolescence is associated with a better overall cardiometabolic profile, assessed using a CMR score, in early adulthood (24 year olds). A cross-sectional analysis of 402 Finnish 6–8-year-old children within the PANIC study found no evidence that a MDP score was related to a CMR score, which is in line with the lack of evidence of association we observed for age 7 diets when considered in isolation. However, a higher score on a Finnish Children Healthy index was associated with reduced CMR score in the boys in that study [27]. The Northern Ireland Young Hearts Study included participants aged 12–15 years followed up at 20–25 years and did not observe any longitudinal relationship between changes in a MDP score and changes in individual CMR risk factors during this period [17]. Our analysis did not look at changes in diet or CMR directly for ease of interpretation considering the multiple exposure and outcome measurements. A cross-sectional analysis of 363 Mediterranean 12–17-year-olds found that a higher adherence to the MDP was associated with decreased prevalence of MetS, characterised by high triacylglycerol levels, hypertension, low HDL-c and central obesity [24]. Differences between these studies in the construction of the MDP scores, the population specific food intakes within each study, the different ages at which dietary patterns and CMR factors were examined, sample sizes and study design may partly explain these discrepancies.

Related to this, we did not find any association between the C-rMED measured at 7, 10 or 13 years and CMR score at 17 years. It is possible that the conventional cardiometabolic risk markers we assessed had not developed into clinically relevant values by this still relatively young age. Indeed, the variation in the values of the cardiometabolic risk measurements (except FMI) was lower at 17 years compared to 24 years (data not shown) which could mean that our study was underpowered to detect an association at the earlier age. This is in line with previous findings from ALSPAC, which showed that a posteriori dietary patterns measured in childhood were not related to cardiovascular risk factors at 17 years, except for BMI [54]. However, in our study the same analysis was repeated at age 24 years, when variation in CMR measures was increased, and there was evidence of an association with the dietary pattern measured at 13 years.

Potential explanations on why we did not observe an association between the C-rMED measured at 7 or 10 years and CMR score at 24 years may be due to varying trends and stability of dietary habits across time. Dietary patterns have been shown to track moderately throughout childhood [15, 55, 56] and our study also found moderate tracking of the C-rMED from 7 to 13 years. However, the Young Finns Study showed that tracking was stronger among participants who were adolescents at baseline compared to tracking starting from younger ages [2, 13]. The period from 7 to 13 years covers the transition into adolescence and the move from primary to secondary school, which often brings shifts in dietary habits partly due to increased autonomy in food choices outside the home [57, 58]. Therefore, in our cohort the dietary patterns assessed at 13 years may have been more representative of the dietary habits over the subsequent decade, compared to dietary patterns measured earlier in childhood. Nevertheless, there was evidence of an additive effect of having a higher C-rMED from 7 to 13 years on the CMR score at 24 years, suggesting a potentially weak association between the CMR score and C-rMED at 7 and 10 years which was only detectable when the cumulative exposure throughout childhood was measured.

In terms of the individual cardiometabolic risk factors, the C-rMED at 13 years predominantly affected anthropometric measures (FMI, BMI and waist circumference) and glucose metabolism (insulin and HOMA-IR) at 13 years. The C-rMED at 7 and 10 years was also related to a decreased odds of larger waist circumference at 24 years. These results support previous cross-sectional and longitudinal studies showing that the MDP has a favourable influence on obesity markers in adults [59] and children [26, 52, 60]. In an international study of European children [26] high scores on a food-frequency-based Mediterranean Diet Score was associated with a 15% reduced odds of overweight/obesity (OR 0.85, 95% CI 0.77, 0.94), which is comparable to our study’s findings for ≥ 80th percentile of BMI (OR 0.83, 95% CI 0.75, 0.92 per 2-unit increase in C-rMED). This is particularly relevant for dietary preventative measures given the huge burden of paediatric obesity worldwide [61]. In addition, obesity has been found to persist from childhood to adulthood and adult obesity is a key risk factor of cardiovascular disease incidence [62].

Our finding that participants with a high C-rMED at 13 years were less likely to have elevated insulin levels and high HOMA-IR scores is also in line with research demonstrating its role in improving glucose metabolism [18, 19, 22]. A 16-week dietary intervention in 49 obese children found the MDP resulted in greater reduction in glucose concentrations, along with other metabolic markers, compared to the control diet [25]. Although there was no evidence of an association with the remaining CMR factors in our study, the ORs were mainly in the postulated/favourable direction. Given the gradual development of indicators of high CMR, a longer-term follow-up, or a larger sample size may be needed to determine if there are any additional associations.

To explore which food groups within the C-rMED at 13 years contributed most to the beneficial effect on the CMR score at 24 years we alternately excluded each component from the C-rMED, and this resulted in minimal changes to the odds of having a high CMR score. However, these analyses suggested that vegetables and cereals made a greater contribution to the observed association. This is consistent with evidence showing that plant-based diets can improve cardiometabolic health in children [13, 63]. There are multiple potential mechanisms that can explain how a predominantly plant-based diet, such as the MDP, can ameliorate insulin resistance, lower blood pressure, improve blood lipids and prevent excess weight gain [19, 22, 64]. These are related to the MDP’s high content of complex carbohydrates, fibre, vitamins and minerals (including non-haem iron, potassium and magnesium), antioxidants, phytochemicals, plant sterols and prebiotics, vegetable protein, healthy fatty acids profile and lower energy–density compared to ‘Western’ diets [63]. These nutritional qualities have been shown to protect against systemic oxidative stress and chronic inflammation, increase satiety, reduce dietary glycaemic load and have favourable effects on gut microbiota [19, 64].

With regards to the study’s limitations, loss to follow-up is a widespread problem in long-term observational studies, especially with repeated data collections and from different sources during follow-up. Although at birth the children in this cohort were relatively representative of the population in the area [30], the notable attrition during the 17-year follow-up produced some loss to follow-up bias. Participants with incomplete dietary data were more likely to be from lower social classes, have less educated mothers, and a worse cardiometabolic profile at 17 years. In addition, research in ALSPAC has shown that dietary patterns correlate with several socioeconomic factors [65], therefore, it is likely that children with less healthy dietary patterns were under-represented in the sample. Accordingly, the children with incomplete dietary and covariate data had lower mean C-rMED scores at 7, 10 and 13 years compared to children with complete data. This may affect the generalisability of the study’s findings to the overall population. Attrition bias due to incomplete covariate data was minimised using multiple imputation of missing data for the main analysis, which prevented further reductions in the final sample size and helped maintain study power. In addition, confounding by socioeconomic indicators was addressed by adjusting for maternal education, family social class and maternal age at delivery. Collider bias could still be a limitation though, since complete dietary and CMR data were both associated with data missingness. Therefore, caution should be taken when interpreting the size of the association observed. The use of self-reported diet diaries to assess children’s dietary intake inevitably introduces some reporting error and bias, although diet diaries are generally less prone to misreporting than food frequency questionnaires [66]. In addition, we did not have 3 days of diet diary records for all the children (a maximum of 1 or 2 days of diet diaries were available for 11.6, 13.6 and 17.4% of the children at 7, 10 and 13 years, respectively). However, we adjusted for number of days of diet diary collection and in additional sensitivity analysis when excluding participants without complete 3-day diet diaries from the complete-case analysis there were minimal changes (data not shown). Validity of dietary reporting was also adjusted for in all models to account for dietary misreporting, along with other potential confounders. However, we cannot rule out residual confounding due to measurement error in these data, or other (unknown) confounding factors not included. Due to the number of analyses carried out to explore which separate factors within the CMR score were driving any association with the C-rMED, there is always the possibility that some of the associations observed were chance findings.

The construction of the C-rMED has its own limitations, since it is based on the participants’ range of food intakes and, therefore, a ‘high’ C-rMED is relative to the distribution of intakes within this study and it may not represent all key elements of the Mediterranean diet. However, the advantage is that is can be readily utilized and adapted to different age groups and populations to give an indication of dietary patterns which were closer to a MDP. It was also a suitable index for the type of dietary data available in our cohort at these ages (daily intakes calculated from diet diaries). In contrast, the Mediterranean Diet Quality Index for children and adolescents (KIDMED) [40] was less compatible with our data because it is based on the weekly/daily consumption of foods/food groups and also certain dietary habits which were not available in our dataset. While olive oil plays central role in the traditional Mediterranean diet, we used a lipid ratio instead because olive oil was not commonly consumed when the dietary data were collected. The lipid ratio has been frequently used within MDP indices constructed in non-Mediterranean countries [42]. It should be noted though that a major source of MUFAs in UK children is from meat and meat products [67]. In contrast, in Mediterranean countries approximately 40% of total MUFA intake is from olive oil [68], which has been well documented for its benefits on cardiometabolic health [18–21].

Both the C-rMED and CMR score are limited by the fact that they are the sum of several components and equal weights are assigned to the components assuming similar importance within the scores. However, the different foods within the C-rMED may not have equivalent effects on the CMR factors and the different risk factors within the CMR score may not have equivalent importance on risk of future cardiometabolic diseases. Furthermore, a direct interpretation of the observed effect size, in terms of extent of clinical benefit, is difficult as the original data was transformed to calculate the CMR score. The 80th percentile cut-offs used to access individual CMR factors do not take account of the different spread in values between the risk factors. Finally, due to the relatively young study sample, it was not possible to assess the associations between childhood MDP and hard cardiovascular disease endpoints, although future research in this cohort will investigate how dietary patterns, including the C-rMED, are related to markers of vascular structure and function (using intima-media thickness and pulse-wave velocity) and inflammatory biomarkers.

This study’s major strength is its prospective design, spanning 17 years of follow-up (from childhood into adulthood) of almost 2000 children. We were able to assess dietary exposure at 3 time points which is advantageous because dietary habits are still evolving during childhood and adolescence [56] and there may be critical time point(s) when dietary habits are more important for future cardiometabolic health. In addition, we were able to investigate the accumulative effect of a Mediterranean-style diet pattern throughout this period of childhood on overall cardiometabolic health. CMR was assessed at two time points (17 and 24 years), which allowed us to examine if the potential influence of this dietary pattern on cardiometabolic health was evident from late adolescence or early adulthood. The use of dietary pattern analysis over single food or nutrient analyses means we were able to assess the effect of combinations of multiple foods/nutrients that can correlate, interact and create a cumulative burden for disease that is not simply the sum of its parts [10]. It can also capture the effects of groups of foods/nutrients whose individual health effect may be undetectable alone. Findings from research on dietary patterns may be more applicable to inform dietary recommendations as people generally find recommendations on cohesive dietary patterns easier to understand and follow than single foods or nutrients. In line with this and our findings, the Council of the European Union report on Nutrition and Physical activity [69] and USA Dietary Guidelines [70] have cited the Mediterranean diet as an example of a healthy eating pattern to follow.

In conclusion, our research showed that in a cohort of children born in the UK in the 1990s, those who had a dietary pattern closer to a Mediterranean-style diet during early adolescence were less likely to have a cluster of CMR factors in their mid-20 s. This could potentially translate into a lower risk of developing overt cardiometabolic disease endpoints later in life, but longer follow-up would need to corroborate this. Our results support previous research advocating the key attributes of the Mediterranean diet (a predominantly plant-based dietary pattern using minimally processed foods and rich in mono- and poly-unsaturated fats) as an important preventative strategy for cardiometabolic diseases, especially if these healthy dietary habits are established and maintained early in life.

## Supplementary Information

Below is the link to the electronic supplementary material.

Supplementary file1 (DOCX 277 KB)

## References

[1] Huang RC, Prescott SL, Godfrey KM, Davis EA (2015). Assessment of cardiometabolic risk in children in population studies: underpinning developmental origins of health and disease mother-offspring cohort studies. J Nutr Sci.

[2] Juonala M, Viikari JS, Raitakari OT (2013). Main findings from the prospective Cardiovascular Risk in Young Finns Study. Curr Opin Lipidol.

[3] Hardy R, Lawlor DA, Kuh D (2015). A life course approach to cardiovascular aging. Future Cardiol.

[4] Berenson G (2002). Childhood risk factors predict adult risk associated with subclinical cardiovascular disease. The Bogalusa Heart Study. Am J Cardiol.

[5] Guardamagna O, Abello F, Cagliero P, Lughetti L (2012). Impact of nutrition since early life on cardiovascular prevention. Ital J Pediatr.

[6] Saeedi P, Shavandi A, Skidmore PML (2019). What do we know about diet and markers of cardiovascular health in children: a review. Int J Environ Res Public Health.

[7] Mente A, de Koning L, Shannon H, Anand S (2009). A systematic review of the evidence supporting a causal link between dietary factors and coronary heart disease. Arch Intern Med.

[8] Mozaffarian D (2016). Dietary and policy priorities for cardiovascular disease, diabetes, and obesity: a comprehensive review. Circulation.

[9] Lazarou C, Newby PK (2011). Use of dietary indexes among children in developed countries. Adv Nutr.

[10] Rocha NP, Milagres LC, Longo GZ, Ribeiro AQ, Novaes JF (2017). Association between dietary pattern and cardiometabolic risk in children and adolescents: a systematic review. J Pediatr (Rio J).

[11] Funtikova AN, Navarro E, Bawaked RA, Fito M, Schroder H (2015). Impact of diet on cardiometabolic health in children and adolescents. Nutr J.

[12] Kaikkonen JE, Mikkila V, Magnussen CG, Juonala M, Viikari JS (2013). Does childhood nutrition influence adult cardiovascular disease risk?–insights from the Young Finns Study. Ann Med.

[13] Mikkila V, Rasanen L, Raitakari OT, Marniemi J, Pietinen P (2007). Major dietary patterns and cardiovascular risk factors from childhood to adulthood. The Cardiovascular Risk in Young Finns Study. Br J Nutr.

[14] Juonala M, Jarvisalo MJ, Maki-Torkko N, Kahonen M, Viikari JS (2005). Risk factors identified in childhood and decreased carotid artery elasticity in adulthood: the Cardiovascular Risk in Young Finns Study. Circulation.

[15] Appannah G, Pot GK, Huang RC, Oddy WH, Beilin LJ (2015). Identification of a dietary pattern associated with greater cardiometabolic risk in adolescence. Nutr Metab Cardiovasc Dis.

[16] Sijtsma FP, Meyer KA, Steffen LM, Van Horn L, Shikany JM (2014). Diet quality and markers of endothelial function: the CARDIA study. Nutr Metab Cardiovasc Dis.

[17] McCourt HJ, Draffin CR, Woodside JV, Cardwell CR, Young IS (2014). Dietary patterns and cardiovascular risk factors in adolescents and young adults: the Northern Ireland Young Hearts Project. Br J Nutr.

[18] Ros E, Martinez-Gonzalez MA, Estruch R, Salas-Salvado J, Fito M (2014). Mediterranean diet and cardiovascular health: teachings of the PREDIMED study. Adv Nutr.

[19] Serra-Majem L, Roman-Vinas B, Sanchez-Villegas A, Guasch-Ferre M, Corella D (2019). Benefits of the Mediterranean diet: epidemiological and molecular aspects. Mol Aspects Med.

[20] Shen J, Wilmot KA, Ghasemzadeh N, Molloy D, Burkman G (2015). Mediterranean dietary patterns and cardiovascular health. Annu Rev Nutr.

[21] Chiva-Blanch G, Badimon L, Estruch R (2014). Latest evidence of the effects of the Mediterranean diet in prevention of cardiovascular disease. Curr Atheroscler Rep.

[22] Tuttolomondo A, Simonetta I, Daidone M, Mogavero A, Ortello A (2019). Metabolic and vascular effect of the Mediterranean diet. Int J Mol Sci.

[23] Casas R, Sacanella E, Urpí-Sardà M, Chiva-Blanch G, Ros E (2014). The effects of the mediterranean diet on biomarkers of vascular wall inflammation and plaque vulnerability. PLoS ONE.

[24] Bibiloni MM, Martínez E, Llull R, Maffiotte E, Riesco M (2011). Metabolic syndrome in adolescents in the Balearic Islands, a Mediterranean region. Nutr Metab Cardiovasc Dis.

[25] Velazquez-Lopez L, Santiago-Diaz G, Nava-Hernandez J, Munoz-Torres AV, Medina-Bravo P (2014). Mediterranean-style diet reduces metabolic syndrome components in obese children and adolescents with obesity. BMC Pediatr.

[26] Tognon G, Hebestreit A, Lanfer A, Moreno LA, Pala V (2014). Mediterranean diet, overweight and body composition in children from eight European countries: cross-sectional and prospective results from the IDEFICS study. Nutr Metab Cardiovasc Dis.

[27] Eloranta AM, Schwab U, Venalainen T, Kiiskinen S, Lakka HM (2016). Dietary quality indices in relation to cardiometabolic risk among Finnish children aged 6–8 years—The PANIC study. Nutr Metab Cardiovasc Dis.

[28] Kamel M, Smith BT, Wahi G, Carsley S, Birken CS (2018). Continuous cardiometabolic risk score definitions in early childhood: a scoping review. Obes Rev.

[29] Fraser A, Macdonald-Wallis C, Tilling K, Boyd A, Golding J (2013). Cohort Profile: the Avon Longitudinal Study of Parents and Children: ALSPAC mothers cohort. Int J Epidemiol.

[30] Boyd A, Golding J, Macleod J, Lawlor DA, Fraser A (2013). Cohort Profile: the 'children of the 90s'–the index offspring of the Avon Longitudinal Study of Parents and Children. Int J Epidemiol.

[31] Golding J, Pembrey M, Jones R, Team AS (2001). ALSPAC–the Avon Longitudinal Study of Parents and Children I. Study methodology. Paediatr Perinat Epidemiol.

[32] Northstone K, Lewcock M, Groom A, Boyd A, Macleod J (2019). The Avon Longitudinal Study of Parents and Children (ALSPAC): an update on the enrolled sample of index children in 2019. Wellcome Open Res.

[33] Harris PA, Taylor R, Thielke R, Payne J, Gonzalez N (2009). Research electronic data capture (REDCap)–a metadata-driven methodology and workflow process for providing translational research informatics support. J Biomed Inform.

[34] Wrieden WL, Longbottom PJ, Adamson AJ, Ogston SA, Payne A (2008). Estimation of typical food portion sizes for children of different ages in Great Britain. Br J Nutr.

[35] Food Standards Agency. Composition of foods integrated dataset (CoFID). McCance and Widdowson's composition of foods integrated dataset. https://www.gov.uk/government/publications/composition-of-foods-integrated-dataset-cofid

[36] Ambrosini GL, Emmett PM, Northstone K, Howe LD, Tilling K (2012). Identification of a dietary pattern prospectively associated with increased adiposity during childhood and adolescence. Int J Obes (Lond).

[37] Emmett P (2009). Dietary assessment in the Avon Longitudinal Study of Parents and Children. Eur J Clin Nutr.

[38] Buckland G, Travier N, Cottet V, Gonzalez CA, Lujan-Barroso L (2013). Adherence to the mediterranean diet and risk of breast cancer in the European prospective investigation into cancer and nutrition cohort study. Int J Cancer.

[39] Willett W, Howe G, Kushi L (1997). Adjustment for total energy intake in epidemiologic studie. Am J Clin Nutr.

[40] Serra-Majem L, Ribas L, Ngo J, Ortega RM, Garcia A (2004). Food, youth and the Mediterranean diet in Spain. Development of KIDMED, Mediterranean Diet Quality Index in children and adolescents. Public Health Nutr.

[41] Buckland G, Gonzalez CA, Agudo A, Vilardell M, Berenguer A (2009). Adherence to the Mediterranean diet and risk of coronary heart disease in the Spanish EPIC Cohort Study. Am J Epidemiol.

[42] Trichopoulou A, Orfanos P, Norat T, Bueno-de-Mesquita B, Ocke MC (2005). Modified Mediterranean diet and survival: EPIC-elderly prospective cohort study. BMJ.

[43] Sesso H, Stampfer M, Rosner B, Hennekens C, Gaziano J (2000). Systolic and diastolic blood pressure, pulse pressure, and mean arterial pressure as predictors of cardiovascular disease risk in men. Hypertension.

[44] Matthews D, Hosker J, Rudenski A, Naylor B, Treacher D (1985). Homeostasis model assessment: insulin resistance and beta-cell function from fasting plasma glucose and insulin concentrations in man. Diabetologia.

[45] Office for National Statistics. The National Statistics Socio-economic classification (NS-SEC). https://www.ons.gov.uk/methodology/classificationsandstandards/otherclassifications/thenationalstatisticssocioeconomicclassificationnssecrebasedonsoc2010. Accessed 21 June 2021

[46] Frysz M, Howe LD, Tobias JH, Paternoster L (2018). Using SITAR (SuperImposition by Translation and Rotation) to estimate age at peak height velocity in Avon Longitudinal Study of Parents and Children. Wellcome Open Res.

[47] Mattocks C, Ness A, Leary S, Tilling K, Blair SN (2008). Use of accelerometers in a large field-based study of children: protocols, design issues, and effects on precision. J Phys Act Health.

[48] Hosmer DW, Lemeshow JS, Sturdivant R (2013) Applied logistic regression, Third Edition. Book Series: Wiley Series in Probability and Statistics. 2013 John Wiley & Sons, Inc.,

[49] Sterne JA, White IR, Carlin JB, Spratt M, Royston P (2009). Multiple imputation for missing data in epidemiological and clinical research: potential and pitfalls. BMJ.

[50] Crawley E, Hughes R, Northstone K, Tilling K, Emond A (2012). Chronic disabling fatigue at age 13 and association with family adversity. Pediatrics.

[51] Little R, Rubin D (2002). Statistical analysis with missing data.

[52] Lydakis C, Stefanaki E, Stefanaki S, Thalassinos E, Kavousanaki M (2012). Correlation of blood pressure, obesity, and adherence to the Mediterranean diet with indices of arterial stiffness in children. Eur J Pediatr.

[53] van de Laar RJ, Stehouwer CD, van Bussel BC, Prins MH, Twisk JW (2013). Adherence to a Mediterranean dietary pattern in early life is associated with lower arterial stiffness in adulthood: the Amsterdam Growth and Health Longitudinal Study. J Intern Med.

[54] Bull CJ, Northstone K (2016). Childhood dietary patterns and cardiovascular risk factors in adolescence: results from the Avon Longitudinal Study of Parents and Children (ALSPAC) cohort. Public Health Nutr.

[55] Northstone K, Emmett PM (2008). Are dietary patterns stable throughout early and mid-childhood? A birth cohort study. Br J Nutr.

[56] Madruga SW, Araujo CL, Bertoldi AD, Neutzling MB (2012). Tracking of dietary patterns from childhood to adolescence. Rev Saude Publica.

[57] Fitzgerald A, Heary C, Nixon E, Kelly C (2010). Factors influencing the food choices of Irish children and adolescents: a qualitative investigation. Health Promot Int.

[58] Lien N, Lytle LA, Klepp KI (2001). Stability in consumption of fruit, vegetables, and sugary foods in a cohort from age 14 to age 21. Prev Med.

[59] Buckland G, Bach A, Serra-Majem L (2008). Obesity and the Mediterranean diet: a systematic review of observational and intervention studies. Obes Rev.

[60] Schröder H, Mendez M, Ribas-Barba L, Covas MI, Serra-Majem L (2010). Mediterranean diet and waist circumference in a representative national sample of young Spaniards. Int J Pediatr Obes.

[61] Di Cesare M, Soric M, Bovet P, Miranda JJ, Bhutta Z (2019). The epidemiological burden of obesity in childhood: a worldwide epidemic requiring urgent action. BMC Med.

[62] Simmonds M, Burch J, Llewellyn A, Griffith C, Yang H (2015). The use of measures of obesity in childhood for predicting obesity and the development of obesity-related diseases in adulthood: a systematic review and meta-analysis. Health Technol Assess.

[63] Desmond MA, Sobiecki J, Fewtrell M, Wells JCK (2018). Plant-based diets for children as a means of improving adult cardiometabolic health. Nutr Rev.

[64] Tosti V, Bertozzi B, Fontana L (2018). Health benefits of the Mediterranean diet: metabolic and molecular mechanisms. J Gerontol A Biol Sci Med Sci.

[65] Northstone K, Smith AD, Cribb VL, Emmett PM (2014). Dietary patterns in UK adolescents obtained from a dual-source FFQ and their associations with socio-economic position, nutrient intake and modes of eating. Public Health Nutr.

[66] Bush LA, Hutchinson J, Hooson J, Warthon-Medina M, Hancock N (2019). Measuring energy, macro and micronutrient intake in UK children and adolescents: a comparison of validated dietary assessment tools. BMC Nutr.

[67] Pot GK, Prynne CJ, Roberts C, Olson A, Nicholson SK (2012). National Diet and Nutrition Survey: fat and fatty acid intake from the first year of the rolling programme and comparison with previous surveys. Br J Nutr.

[68] Linseisen J, Welch AA, Ocke M, Amiano P, Agnoli C (2009). Dietary fat intake in the European Prospective Investigation into Cancer and Nutrition: results from the 24-h dietary recalls. Eur J Clin Nutr.

[69] The Council of the European Union (2014) Council conclusions on nutrition and physical activity. Employment, Social Policy, Health and Consumer Affairs Council Meeting. https://www.consilium.europa.eu/uedocs/cms_data/docs/pressdata/en/lsa/143285.pdf. Accessed June 2021

[70] US Department of Health and Human Services (2015). Dietary Guidelines for Americans 2015–2020. Eighth Edition. https://health.gov/our-work/food-nutrition/previous-dietary-guidelines/2015. Accessed June 2021

