# Supplementary material for: Prospective association between a Mediterranean-style dietary score in childhood and cardiometabolic risk in young adults from the ALSPAC birth cohort
Source: Eur J Nutr. 2021 Sep 17;61(2):737–52. doi: 10.1007/s00394-021-02652-7 (PMC8854247; doi:10.1007/s00394-021-02652-7)
Supplement: Supplementary file 1 — Supplementary file1 (DOCX 277 KB) [file 394_2021_2652_MOESM1_ESM.docx]

**Supplementary Material**

**European Journal of Nutrition**

**Prospective association between a Mediterranean-style dietary score in childhood and cardiometabolic risk in young adults from the ALSPAC birth cohort.**

Buckland G^*^, Taylor CM, Emmett PM, Johnson L, Northstone K

**^*^Centre for Academic Child Health, Bristol Medical School, University of Bristol, Bristol, UK**

**g.buckland@bristol.ac.uk**

**Appendix I**

The distribution of participants from the Avon Longitudinal Study of Parents and Children (ALSPAC), according to completeness of dietary data at 7, 10 and 13 years, Cardiometabolic Risk (CMR) score data at 17 years and 24 years and covariate data, in the observed (A) and imputed (B) datasets.

**
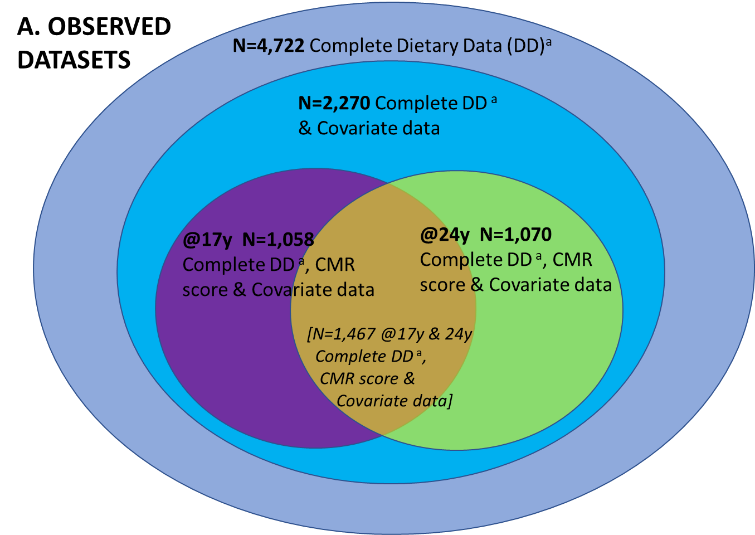
**

**
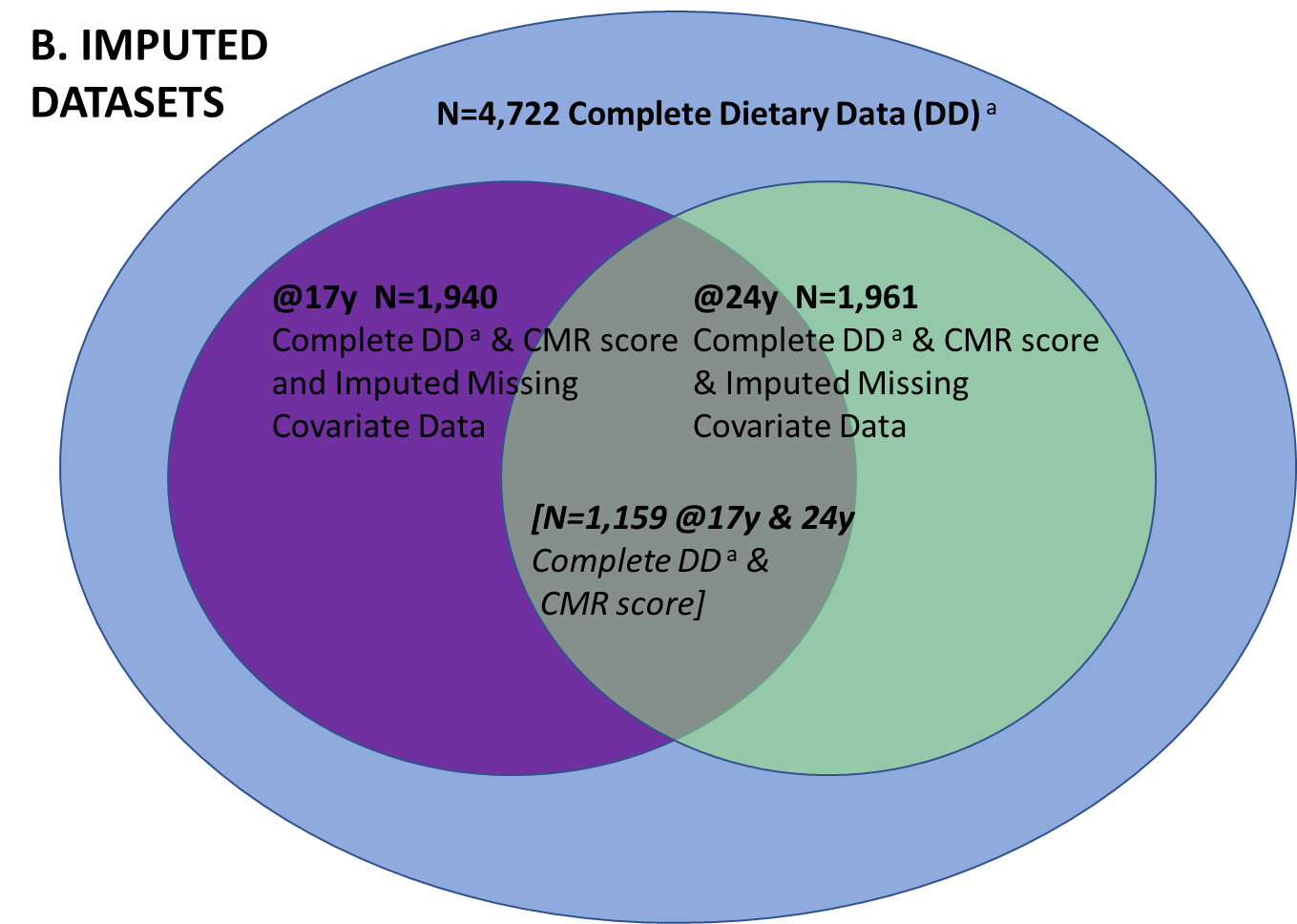
**

^a^Complete Dietary Data (DD) refers to dietary data collected from ≥1 diet diary at all three ages (7 years, 10 years and 13 years).

**Appendix II**

Data missingness for covariates in ALSPAC@17 and ALSPAC@24

| **Covariates included in logistic regression models (excluding exposure and outcome)** | **ALSPAC @ 17** | **ALSPAC @ 24** |
| --- | --- | --- |
|  | **n=1,940** | **n=1,961** |
|  | **Missing observations n (%)** | **Missing observations n (%)** |
| Sex | 0 (0%) | 0 (0%) |
| Age at 7 years | 0 (0%) | 0 (0%) |
| Age at 10 years | 0 (0%) | 0 (0%) |
| Age at 13 years | 0 (0%) | 0 (0%) |
| Energy intake at 7 years | 0 (0%) | 0 (0%) |
| Energy intake at 10 years | 0 (0%) | 0 (0%) |
| Energy intake at 13 years | 0 (0%) | 0 (0%) |
| Number of days diet diary collected at 7 years | 0 (0%) | 0 (0%) |
| Number of days diet diary collected at 10 years | 0 (0%) | 0 (0%) |
| Number of days diet diary collected at 13 years | 0 (0%) | 0 (0%) |
| Validity of dietary reporting at 7 years | 9 (0.5) | 8 (0.4) |
| Validity of dietary reporting at 10 years | 7 (0.4) | 5 (0.3) |
| Validity of dietary reporting at 13 years | 11 (0.6) | 9 (0.5) |
| Gestational age | 73 (3.8) | 76 (3.9) |
| Maternal age at delivery | 73 (3.8) | 76 (3.9) |
| Birth weight | 100 (5.2) | 101 (5.2) |
| Maternal pre-pregnancy BMI | 201 (10.4) | 196 (10.0) |
| Puberty timing | 1 (0.1) | 72 (3.7) |
| Maternal education | 100 (5.2) | 100 (5.1) |
| Highest family social class | 146 (7.5) | 143 (7.3) |
| Physical Activity (MVPA) at 13 years | 411 (21.2) | 417 (21.3) |

| **Appendix III.**  Comparison of baseline characteristics and cardiometabolic risk factors at 17 years and 24 years in ALSPAC participants who had complete dietary and covariate data at all three ages (7, 10 and 13 years) and those who did not**.** | | | | | | |
| --- | --- | --- | --- | --- | --- | --- |
|  |  |  |  |  |  |  |
| **Characteristics of the ALSPAC index children** | **Eligible ALSPAC cohort** | | | | | |
|  |  | **Incomplete dietary and covariate data^a^** | | **Complete dietary and covariate data^a^** | | **P-value^c^** |
|  | **N total** | **N** | **% or mean±sd^b^** | **N** | **% or mean±sd^b^** |  |
| ***Baseline Characteristics*** |  |  |  |  |  |  |
| *Sex* |  |  |  |  |  |  |
| Male | 7,596 | 6,577 | 52.2 | 1,019 | 44.9 |  |
| Female | 7,277 | 6,026 | 47.8 | 1,251 | 55.1 | <0.001 |
| BMI at 10 years, kg/m^2^ | 7,457 | 5,199 | 18.4 ± 3.2 | 2,258 | 18.0 ± 2.9 | <0.001 |
| Energy intake at 10 years, kJ/d | 7,453 | 5,183 | 7,747.8 ± 1,659.3 | 2,270 | 7,872.2 ±1,526.7 | <0.001 |
| C-rMED at 7 years | 7,274 | 5,004 | 6.7 ± 2.3 | 2,270 | 6.9 ± 2.3 | <0.001 |
| C-rMED at 10 years | 7,460 | 5,190 | 6.4 ± 2.3 | 2,270 | 6.9 ± 2.3 | <0.001 |
| C-rMED at 13 years | 6,099 | 3,829 | 6.3 ± 2.4 | 2,270 | 6.7 ± 2.4 | <0.001 |
| Maternal age at delivery, years | 13,961 | 11,691 | 27.7 ± 5.0 | 2,270 | 29.6 ± 4.2 | <0.001 |
| Maternal pre-pregnancy BMI, kg/m^2^ | 11,520 | 9,250 | 23.0 ± 4.0 | 2,270 | 22.7 ± 3.5 | <0.001 |
| Maternal highest education |  |  |  |  |  |  |
| CSE, Vocational or O level | 8,018 | 6,906 | 68.2 | 1,112.0 | 49 |  |
| A-level or Degree | 4,384 | 3,226 | 31.8 | 1,158.0 | 51 | <0.001 |
| Highest household social class |  |  |  |  |  |  |
| I and II | 2,983 | 2,230 | 24.3 | 753 | 33.2 |  |
| III, IV and V | 8,474 | 6,957 | 75.7 | 1,517 | 66.8 | <0.001 |
| ***CMR factors at 17 years*** |  |  |  |  |  |  |
| Fat Mass Index, kg/m^2^ | 4,825 | 3,056 | 6.5 ± 3.9 | 1,769 | 6.1 ± 3.5 | 0.013 |
| HDL cholesterol, mmol/L | 3,285 | 2,090 | 1.3 ± 0.3 | 1,195 | 1.3 ±0.3 | 0.030 |
| LDL cholesterol, mmol/L | 3,285 | 2,090 | 2.1 ± 0.6 | 1,195 | 2.1 ± 0.6 | 0.424 |
| Triacylglycerol, mmol/L | 3,285 | 2,090 | 0.8 ± 0.4 | 1,195 | 0.8 ± 0.3 | 0.100 |
| Mean arterial blood pressure, mmHg | 4,657 | 2,939 | 81.8 ± 6.3 | 1,718 | 81.2 ± 5.9 | 0.012 |
| HOMA-IR | 3,215 | 2,049 | 2.0 ± 2.4 | 1,166 | 1.8 ± 2.0 | 0.015 |
| ***CMR factors at 24 years*** |  |  |  |  |  |  |
| Fat Mass Index, kg/m^2^ | 3,850 | 2,462 | 0.8 ± 0.4 | 1,388 | 0.8 ± 0.4 | 0.015 |
| HDL cholesterol, mmol/L | 3,252 | 2,095 | 1.5 ± 0.4 | 1,157 | 1.6 ± 0.4 | 0.284 |
| LDL cholesterol, mmol/L | 3,250 | 2,094 | 2.4 ± 0.8 | 1,156 | 2.5 ± 0.8 | 0.646 |
| Triacylglycerol, mmol/L | 3,251 | 2,095 | 1.0 ± 0.6 | 1,156 | 1.0 ± 0.5 | 0.765 |
| Mean arterial blood pressure, mmHg | 3,994 | 2,571 | 83.3 ± 8.1 | 1,423 | 83.3 ± 8.3 | 0.978 |
| HOMA-IR | 3,252 | 2,095 | 2.3 ± 2.4 | 1,157 | 2.4 ± 3.2 | 0.723 |
| Abbreviations: CMR; Cardiometabolic Risk. HOMA-IR; Homeostatic Model Assessment of Insulin Resistance. | | | | | | |
| ^a^Incomplete dietary and covariate data refers to missing dietary data at ≥1 age (7, 10, 13 years) and missing data on any of the following covariates: dietary misreporting, birthweight, gestational age, puberty stage, physical activity at 13years, pre-pregnancy BMI of mother, age of mother at delivery, mother's highest education level, highest family social class. Complete dietary and covariate data refers to dietary data collected at all three ages (7, 10 and 13 years) and complete data on the covariates listed above. | | | | | | |
| ^b^Percentage (all such values and refers to column percentages) or Mean ± SD (all such values) | | | | | |  |
| ^c^Chi-squared test for categorical variables and Kruskal-Wallis test for continuous variables | | | | |  |  |

| **Appendix IV**  Comparison of baseline characteristics in ALSPAC participants with imputed and observed data. | | | | | | |
| --- | --- | --- | --- | --- | --- | --- |
|  |  |  |  |  |  |  |
| **Characteristics of the ALSPAC index children** | **Dataset with outcome at 17 years (n=1,940)** | | | **Dataset with outcome at 24 years (n=1,961)** | | |
|  | **Imputed** | **Observed** | **% of data imputed** | **Imputed** | **Observed** | **% of data imputed** |
|  | **Mean ± SE or %** | |  | **Mean ± SE or %** | |  |
| **Birth weight, grams** | 3,431 ± 13.4 | 3,441 ± 12.5 | 5.2 | 3,418.2 ± 13.1 | 3,421.5 ± 12.4 | 5.2 |
| **Maternal pre-pregnancy BMI, kg/m^2^** | 22.7 ± 0.1 | 22.7 0.1 | 10.4 | 22.6 ± 0.1 | 22.6 ± 0.1 | 10.0 |
| **Gestational age, months** |  |  |  |  |  |  |
| 28-31 | 0.7 | 0.6 |  | 0.6 | 0.5 |  |
| 32-36 | 4.7 | 4.5 |  | 4.7 | 4.6 |  |
| 37-41 | 88.7 | 88.9 | 3.8 | 87.7 | 88.0 | 3.9 |
| 42-47 | 5.9 | 6.0 |  | 7.0 | 7.0 |  |
| **Maternal age at delivery, years** |  |  |  |  |  |  |
| <16-24 | 11.6 | 11.3 |  | 10.1 | 9.9 |  |
| 25-29 | 38.2 | 38.0 |  | 38.6 | 38.4 |  |
| 30-34 | 35.0 | 35.4 | 3.8 | 36.5 | 36.8 | 3.9 |
| ≥35 | 15.2 | 15.4 |  | 14.8 | 14.9 |  |
| **Maternal highest education** |  |  |  |  |  |  |
| Certificate of Secondary Education | 7.8 | 7.8 |  | 6.8 | 6.9 |  |
| Vocational training | 7.2 | 6.4 |  | 5.5 | 5.4 |  |
| O-level | 32.2 | 32.2 | 5.2 | 34.2 | 33.5 | 5.1 |
| A-level | 30.6 | 31.0 |  | 30.3 | 30.6 |  |
| Degree or higher | 22.1 | 22.6 |  | 23.1 | 23.5 |  |
| **Highest household social class** |  |  |  |  |  |  |
| I | 5.0 | 5.1 |  | 5.8 | 5.8 |  |
| II | 30.3 | 30.4 |  | 30.2 | 30.6 |  |
| III non-manual | 28.3 | 28.7 | 7.5 | 28.3 | 28.5 | 7.3 |
| III manual | 22.6 | 22.5 |  | 23.2 | 22.9 |  |
| IV and V | 13.7 | 13.3 |  | 12.6 | 12.2 |  |
| **Puberty timing** |  |  |  |  |  |  |
| Early | 51.5 | 51.5 |  | 50.7 | 50.7 |  |
| Late | 48.5 | 48.5 | 0.1 | 49.3 | 49.3 | 3.7 |
| **Accuracy of dietary reporting - 7 years** |  |  |  |  |  |  |
| Under-reporting | 9.3 | 9.3 |  | 10.0 | 10.4 |  |
| Accurate reporting | 75.7 | 75.7 | 0.5 | 76.3 | 76.3 | 0.4 |
| Over-reporting | 15.0 | 15.0 |  | 13.6 | 13.6 |  |
| **Accuracy of dietary reporting - 10 years** |  |  |  |  |  |  |
| Under-reporting | 30.2 | 30.2 |  | 32.7 | 32.7 |  |
| Accurate reporting | 67.1 | 67.1 | 0.4 | 64.5 | 64.5 | 0.3 |
| Over-reporting | 2.7 | 2.7 |  | 2.9 | 2.9 |  |
| **Accuracy of dietary reporting - 13 years** |  |  |  |  |  |  |
| Under-reporting | 60.0 | 60.1 |  | 60.2 | 60.2 |  |
| Acurate and over-reporting | 40.0 | 39.9 | 0.6 | 39.8 | 39.8 | 0.5 |
| **Moderate-to-vigorous physical activity** |  |  |  |  |  |  |
| <20 mins | 47.0 | 47.9 |  | 49.6 | 50.6 |  |
| ≥20 to <40 mins | 36.3 | 36.2 | 21.2 | 35.2 | 35.1 | 21.3 |
| 40 to <60 mins | 11.4 | 11.1 |  | 10.8 | 10.4 |  |
| 60 mins | 5.3 | 4.9 |  | 4.4 | 4.0 |  |

**Appendix V.** Adjusted OR and 95% CI for the association between the children’s relative Mediterranean diet score (C-rMED) at 7, 10 and 13 years and scoring ≥80th percentile on cardiometabolic risk (CMR) score at 17 and 24 years by sex, using imputed datasets

| **Children's relative Mediterranean diet score (C-rMED)^a^** | **CMR Score (≥80th percentile) at 17 years (n=1,940)** | | | | | | **CMR Score (≥80th percentile) at 24 years (1,961)** | | | | | |
| --- | --- | --- | --- | --- | --- | --- | --- | --- | --- | --- | --- | --- |
|  |  | **Female** | |  | **Male** | |  | **Female** | |  | **Male** | |
|  | **N** | **OR (95%CI)** | **P-trend** | **N** | **OR (95%CI)** | **P-trend** | **N** | **OR (95%CI)** | **P-trend** | **N** | **OR (95%CI)** | **P-trend** |
| ***C-rMED at 7 years*** |  |  |  |  |  |  |  |  |  |  |  |  |
| Low | 286 | Reference |  | 308 | Reference |  | 307 | Reference |  | 267 | Reference |  |
| Medium | 439 | 1.06 (0.72, 1.58) |  | 416 | 1.06 (0.72, 1.56) |  | 517 | 1.11 (0.75, 1.63) |  | 373 | 0.60 (0.40, 0.90) |  |
| High | 244 | 0.97 (0.61, 1.56) | 0.940 | 247 | 0.70 (0.44, 1.12) | 0.182 | 291 | 1.21 (0.77, 1.89) | 0.403 | 206 | 0.60 (0.37, 0.98) | 0.024 |
| Per 2-unit increment | 969 | 0.96 (0.83, 1.11) | 0.603 | 971 | 0.89 (0.77, 1.02) | 0.101 | 1115 | 1.06 (0.93, 1.22) | 0.385 | 846 | 0.85 (0.73, 0.99) | 0.039 |
| ***C-rMED at 10 years*** |  |  |  |  |  |  |  |  |  |  |  |  |
| Low | 266 | Reference |  | 300 | Reference |  | 319 | Reference |  | 256 | Reference |  |
| Medium | 452 | 1.41 (0.94, 2.12) |  | 459 | 1.01 (0.69, 1.48) |  | 487 | 0.84 (0.57, 1.23) |  | 391 | 1.03 (0.69, 1.55) |  |
| High | 251 | 1.22 (0.75, 1.97) | 0.396 | 212 | 0.80 (0.48, 1.31) | 0.424 | 309 | 0.92 (0.59, 1.42) | 0.665 | 199 | 0.65 (0.39, 1.11) | 0.155 |
| Per 2-unit increment | 969 | 1.07 (0.93, 1.24) | 0.359 | 971 | 0.94 (0.81, 1.08) | 0.394 | 1115 | 0.99 (0.86, 1.14) | 0.892 | 846 | 0.87 (0.75, 1.01) | 0.066 |
| ***C-rMED at 13 years*** |  |  |  |  |  |  |  |  |  |  |  |  |
| Low | 287 | Reference |  | 374 | Reference |  | 316 | Reference |  | 307 | Reference |  |
| Medium | 428 | 0.69 (0.47, 1.02) |  | 423 | 1.04 (0.72, 1.50) |  | 505 | 0.68 (0.47, 0.99) |  | 377 | 0.80 (0.54, 1.17) |  |
| High | 254 | 0.68 (0.43, 1.08) | 0.086 | 174 | 0.88 (0.53, 1.45) | 0.717 | 294 | 0.72 (0.46, 1.13) | 0.120 | 162 | 0.63 (0.37, 1.05) | 0.065 |
| Per 2-unit increment | 969 | 0.92 (0.80, 1.07) | 0.274 | 971 | 0.97 (0.84, 1.12) | 0.705 | 1115 | 0.90 (0.79, 1.04) | 0.153 | 846 | 0.87 (0.75, 1.01) | 0.062 |
| ***C-rMED tracking 7-10-13 years^b^*** |  |  |  |  |  |  |  |  |  |  |  |  |
| C-rMED low all ages | 66 | Reference |  | 87 | Reference |  | 74 | Reference |  | 60 | Reference |  |
| C-rMED mixed across ages | 306 | 0.87 (0.44, 1.73) |  | 333 | 1.05 (0.55, 2.00) |  | 348 | 0.83 (0.43, 1.58) |  | 297 | 0.86 (0.43, 1.71) |  |
| C-rMED medium at least twice | 416 | 0.95 (0.49, 1.86) |  | 417 | 1.01 (0.54, 1.89) |  | 476 | 0.55 (0.29, 1.06) |  | 367 | 0.65 (0.33, 1.28) |  |
| C-rMED high at least twice | 181 | 0.76 (0.35, 1.62) | 0.635 | 134 | 0.88 (0.41, 1.89) | 0.662 | 217 | 0.60 (0.29, 1.24) | 0.039 | 122 | 0.34 (0.14, 0.79) | 0.003 |
| Abbreviations: C-rMED; Children's relative Mediterranean diet score. CMR score; Cardiometabolic risk score. Adjusted Multivariable regression model adjusted for sex, age at dietary data collection, number of days diet diary collected, dietary misreporting, birthweight, gestational age, puberty stage, physical activity at 13years, pre-pregnay BMI of mother, age of mother at delivery, mother's highest education level, highest family social class. | | | | | | | | | | | | |
| ^a^C-rMED includes eight food components and scores subjects from 0-15 (low (0-5), medium (6-8) and high (9-15)) | | | | | | | | | | | | |
| ^b^C-rMED medium at least twice includes the following combination of C-rMED scores across any of the three age groups (medium+medium+low or medium+medium+medium or medium+medium+high). C-rMED high at least twice includes the following combination of C-rMED scores across any of the three age agroups (high+high+low or high+high+medium or high+high+high). | | | | | | | | | | | | |

| **Appendix VI.** Unadjusted and adjusted OR and 95% CI for the association between the children’s relative Mediterranean diet score (C-rMED) at 7, 10 and 13 years and scoring ≥80th percentile on cardiometabolic risk score at 17 and 24 years, using all available data (complete-case analysis) | | | | | | | | | | |
| --- | --- | --- | --- | --- | --- | --- | --- | --- | --- | --- |
|  |  |  |  |  |  |  |  |  |  |  |
| **Children's relative Mediterranean diet score (C-rMED)^a^** | **CMR Score (≥80th percentile) - 17 years (n=1,058)** | | | | | **CMR Score (≥80th percentile) - 24 years (1,070)** | | | | |
|  |  | **Unadjusted** | | **Adjusted** | |  | **Unadjusted** | | **Adjusted** | |
|  | **N** | **OR (95%CI)** | **P-trend** | **OR (95%CI)** | **P-trend** | **N** | **OR (95%CI)** | **P-trend** | **OR (95%CI)** | **P-trend** |
| ***C-rMED at 7 years*** |  |  |  |  |  |  |  |  |  |  |
| Low | 316 | Reference |  | Reference |  | 303 | Reference |  | Reference |  |
| Medium | 455 | 0.94 (0.67,1.34) |  | 0.97 (0.67,1.41) |  | 471 | 0.77 (0.54,1.10) |  | 0.74 (0.51,1.07) |  |
| High | 287 | 0.65 (0.43,0.98) | 0.048 | 0.70 (0.45,1.10) | 0.137 | 296 | 0.72 (0.49,1.08) | 0.112 | 0.75 (0.49,1.15) | 0.428 |
| Per 2-unit increment | 1058 | 0.91 (0.80,1.04) | 0.158 | 0.93 (0.80,1.08) | 0.326 | 1,070 | 0.93 (0.82,1.06) | 0.271 | 0.93 (0.81,1.07) | 0.329 |
| ***C-rMED at 10 years*** |  |  |  |  |  |  |  |  |  |  |
| Low | 283 | Reference |  | Reference |  | 290 | Reference |  | Reference |  |
| Medium | 506 | 1.07 (0.75,1.54) |  | 1.06 (0.72,1.56) |  | 483 | 0.76 (0.54,1.08) |  | 0.80 (0.55,1.17) |  |
| High | 269 | 0.84 (0.54,1.29) | 0.253 | 0.91 (0.58,1.44) | 0.719 | 297 | 0.55 (0.37,0.84) | 0.005 | 0.63 (0.40,0.98) | 0.041 |
| Per 2-unit increment | 1058 | 0.97 (0.85,1.10) | 0.620 | 1.00 (0.87,1.15) | 0.996 | 1,070 | 0.84 (0.74,0.95) | 0.005 | 0.87 (0.76,1.00) | 0.049 |
| ***C-rMED at 13 years*** |  |  |  |  |  |  |  |  |  |  |
| Low | 342 | Reference |  | Reference |  | 327 | Reference |  | Reference |  |
| Medium | 471 | 1.02 (0.72,1.44) |  | 1.04 (0.72,1.50) |  | 470 | 0.80 (0.57,1.12) |  | 0.83 (0.58,1.20) |  |
| High | 245 | 0.76 (0.49,1.16) | 0.303 | 0.83 (0.53,1.32) | 0.474 | 273 | 0.60 (0.39,0.90) | 0.014 | 0.62 (0.40,0.96) | 0.034 |
| Per 2-unit increment | 1058 | 0.96 (0.90,1.03) | 0.276 | 0.98 (0.91,1.05) | 0.572 | 1,070 | 0.85 (0.75,0.96) | 0.010 | 0.86 (0.75,0.98) | 0.025 |
| ***C-rMED tracking 7-10-13 years^b^*** |  |  |  |  |  |  |  |  |  |  |
| C-rMED low all ages | 76 | Reference |  | Reference |  | 69 | Reference |  | Reference |  |
| C-rMED mixed across ages | 341 | 0.90 (0.49,1.66) |  | 0.96 (0.50,1.84) |  | 344 | 1.08 (0.60,1.97) |  | 1.02 (0.53,1.96) |  |
| C-rMED medium at least twice | 449 | 1.01 (0.55,1.83) |  | 0.98 (0.52,1.85) |  | 447 | 0.67 (0.37,1.21) |  | 0.62 (0.33,1.19) |  |
| C-rMED high at least twice | 192 | 0.72 (0.37,1.41) | 0.473 | 0.75 (0.36,1.54) | 0.435 | 210 | 0.47 (0.24,0.93) | <0.001 | 0.47 (0.22,0.98) | <0.001 |
| Abbreviations: C-rMED; Children's relative Mediterranean diet score. CMR score; Cardiometabolic risk score. Adjusted: Multivariable regression model adjusted for sex, age at dietary data collection, number of days diet diary collected, dietary misreporting, birthweight, gestational age, puberty stage, physical activity at 13years, pre-pregnany BMI of mother, age of mother at delivery, mother's highest education level, highest family social class. | | | | | | | | | | |
| ^a^C-rMED includes eight food components and scores subjects from 0-15 (low (0-5), medium (6-8) and high (9-15)) | | | | | | | | | | |
| ^b^C-rMED medium at least twice includes the following combination of C-rMED scores across any of the three age groups (medium+medium+low or medium+medium+medium or medium+medium+high). C-rMED high at least twice includes the following combination of C-rMED scores across any of the three age groups (high+high+low or high+high+medium or high+high+high). | | | | | | | | | | |

| **Appendix VII. Unadjusted and adjusted ß coefficients and 95% CI for the association between the children’s relative Mediterranean diet score (C-rMED) at 7, 10 and 13 years (per 2-unit increment) and continuous cardiometabolic risk score at 17 and 24 years, using all available data (complete-case analysis)** | | | | | | | | | | |
| --- | --- | --- | --- | --- | --- | --- | --- | --- | --- | --- |
|  |  |  |  |  |  |  |  |  |  |  |
| **Children's relative Mediterranean diet score (C-rMED)^a^** | **CMR Score (continuous) - 17 years (n=1,058)** | | | | | **CMR Score (continuous) - 24 years (1,070)** | | | | |
|  |  | **Unadjusted** | | **Adjusted** | |  | **Unadjusted** | | **Adjusted** | |
|  | **N** | **ß (95%CI)** | **P-trend** | **ß (95%CI)** | **P-trend** | **N** | **ß (95%CI)** | **P-trend** | **ß (95%CI)** | **P-trend** |
| ***C-rMED at 7 years*** |  |  |  |  |  |  |  |  |  |  |
| Per 2-unit increment | 1,058 | -0.03 (-0.06,0.003) | 0.073 | -0.02 (-0.05,0.01) | 0.173 | 1,070 | -0.02 (-0.05,0.01) | 0.223 | -0.02 (-0.05,0.02) | 0.335 |
| ***C-rMED at 10 years*** |  |  |  |  |  |  |  |  |  |  |
| Per 2-unit increment | 1,058 | -0.01 (-0.04,0.02) | 0.342 | 0.00 (-0.03,0.03) | 0.979 | 1,070 | -0.02 (-0.05,0.01) | 0.223 | -0.01 (-0.04,0.02) | 0.686 |
| ***C-rMED at 13 years*** |  |  |  |  |  |  |  |  |  |  |
| Per 2-unit increment | 1,058 | -0.03 (-0.06,-0.001) | 0.045 | -0.02 (-0.05,0.01) | 0.176 | 1,070 | -0.04 (-0.07,-0.01) | 0.008 | -0.03 (-0.06,-0.002) | 0.040 |
| Abbreviations: C-rMED; Children's relative Mediterranean diet score. CMR score; Cardiometabolic risk score. | | | | | | | | | | |
| ^a^C-rMED includes eight food components and scores subjects from 0-15 (low (0-5), medium (6-8) and high (9-15)) | | | | | | | | | | |
| Adjusted: Multivariable regression model adjusted for sex, age at dietary data collection, number of days diet diary collected, dietary misreporting, birthweight, gestational age, puberty stage, physical activity at 13years, pre-pregnancy BMI of mother, age of mother at delivery, mother's highest education level, highest family social class. | | | | | | | | | | |

| **Appendix VIII.** Adjusted OR and 95% CI for the association between the children’s relative Mediterranean diet score (C-rMED) at 7, 10 and 13 years and individual cardiometabolic risk factors at 17 and 24 years, using all available data (complete-case analysis) | | | | | | |
| --- | --- | --- | --- | --- | --- | --- |
|  |  |  |  |  |  |  |
| **CMR Factors: Odds of being above the 80th percentile^a^** | **CMR Factors at 17y (n=1,058)** | | | **CMR Factors at 24y (n=1,070)** | | |
|  | **+2-unit C-rMED 7y** | **+2-unit C-rMED 10y** | **+2-unit C-rMED 13y** | **+2-unit C-rMED 7y** | **+2-unit C-rMED 10y** | **+2-unit C-rMED 13y** |
|  | **OR (95%CI)^b^** | **OR (95%CI)^b^** | **OR (95%CI)^b^** | **OR (95%CI)^b^** | **OR (95%CI)^b^** | **OR (95%CI)^b^** |
| ***Anthropometric*** |  |  |  |  |  |  |
| Body Mass Index | 0.90 (0.78,1.04) | 0.96 (0.83,1.11) | 0.93 (0.80,1.08) | 0.86 (0.74,0.99) | 0.91 (0.79,1.05) | 0.85 (0.74,0.98) |
| Fat Mass Index^c^ | 0.88 (0.76,1.02) | 0.90 (0.78,1.05) | 0.83 (0.71,0.97) | 0.93 (0.81,1.07) | 0.83 (0.72,0.95) | 0.79 (0.69,0.91) |
| Waist circumference | N/A | N/A | N/A | 0.89 (0.77,1.03) | 0.90 (0.78,1.04) | 0.85 (0.74,0.98) |
| ***Blood lipids*** |  |  |  |  |  |  |
| Total cholesterol | 0.93 (0.81,1.06) | 1.08 (0.95,1.24) | 0.90 (0.79,1.03) | 1.06 (0.93,1.21) | 1.03 (0.90,1.16) | 1.06 (0.93,1.20) |
| HDL cholesterol^c^ | 1.02 (0.89,1.16) | 1.00 (0.88,1.15) | 1.01 (0.88,1.16) | 0.95 (0.83,1.09) | 0.94 (0.83,1.08) | 0.91 (0.80,1.04) |
| LDL cholesterol^c^ | 0.96 (0.84,1.10) | 1.00 (0.88,1.14) | 0.96 (0.84,1.10) | 1.05 (0.92,1.19) | 0.93 (0.81,1.05) | 0.99 (0.87,1.12) |
| Triacylglycerol^c^ | 1.12 (0.98,1.28) | 1.03 (0.90,1.17) | 1.07 (0.94,1.22) | 0.98 (0.86,1.12) | 1.00 (0.88,1.14) | 1.01 (0.89,1.15) |
| ***Blood pressure*** |  |  |  |  |  |  |
| Systolic BP | 1.01 (0.87,1.18) | 1.15 (0.99,1.34) | 1.02 (0.88,1.19) | 0.95 (0.82,1.11) | 1.02 (0.88,1.17) | 0.92 (0.80,1.06) |
| Diastolic BP | 0.83 (0.72,0.95) | 0.97 (0.84,1.11) | 0.92 (0.80,1.05) | 0.91 (0.79,1.04) | 1.04 (0.91,1.19) | 0.94 (0.82,1.07) |
| Mean Arterial BP^c^ | 0.88 (0.77,1.01) | 0.91 (0.79,1.05) | 0.90 (0.78,1.03) | 0.95 (0.82,1.09) | 1.08 (0.94,1.23) | 0.90 (0.79,1.03) |
| ***Glucose metabolism*** |  |  |  |  |  |  |
| Insulin | 0.97 (0.85,1.11) | 0.99 (0.87,1.14) | 0.86 (0.75,0.99) | 0.94 (0.82,1.08) | 0.94 (0.82,1.07) | 0.81 (0.71,0.93) |
| Glucose | 1.09 (0.95,1.26) | 1.11 (0.96,1.27) | 1.01 (0.87,1.17) | 1.13 (0.98,1.30) | 1.01 (0.88,1.16) | 1.12 (0.97,1.28) |
| HOMA-IR^c^ | 0.97 (0.85,1.11) | 0.96 (0.84,1.09) | 0.86 (0.75, 0.98) | 0.97 (0.85,1.12) | 0.94 (0.82,1.08) | 0.86 (0.75,0.98) |
| Abbreviations: C-rMED: children's relative Mediterranean diet score; CMR score: Cardiometabolic risk score. HOMA-IR: Homeostatic Model Assessment of Insulin Resistance. BP: Blood Pressure. HDL cholesterol: High-density lipoprotein cholesterol. LDL cholesterol: low-density lipoprotein cholesterol. | | | | | | |
| ^a^Odds of being above the 80th percentile except for HDL cholesterol, which is odds of being below the 20th percentile. ^b^Odds Ratios (OR) derived from multivariable logistic regression models adjusted for sex, age at dietary data collection, number of days diet diary collected, dietary misreporting, birthweight, gestational age, puberty stage, physical activity at 13 years, pre-pregnany BMI of mother, age of mother at delivery, mother's highest education level, highest family social class. ^c^Cardiometabolic parameters included in the Cardiometabolic Risk Score. | | | | | | |
